# Importin-αs are required for the nuclear localization and function of the *Plasmopara viticola* effector PvAVH53

**DOI:** 10.1038/s41438-021-00482-6

**Published:** 2021-03-01

**Authors:** Tingting Chen, Jing Peng, Xiao Yin, Meijie Li, Gaoqing Xiang, Yuejin Wang, Yan Lei, Yan Xu

**Affiliations:** 1 State Key Laboratory of Crop Stress Biology in Arid Areas (Northwest A&F University), Yangling, Shaanxi P.R. China; 2 College of Horticulture, Northwest A&F University, Yangling, Shaanxi P.R. China; 3 Key Laboratory of Horticultural Plant Biology and Germplasm Innovation in Northwest China, Ministry of Agriculture, Yangling, Shaanxi P.R. China; 4 Fruit Research Institute, Fujian Academy of Agricultural Sciences, 350013 Fuzhou, Fujian China

**Keywords:** Plant immunity, Fungi

## Abstract

Plant pathogenic oomycetes deliver a troop of effector proteins into the nucleus of host cells to manipulate plant cellular immunity and promote colonization. Recently, researchers have focused on identifying how effectors are transferred into the host cell nucleus, as well as the identity of the nuclear targets. In this study, we found that the RxLR effector PvAVH53 from the grapevine (*Vitis vinifera*) oomycete pathogen *Plasmopara viticola* physically interacts with grapevine nuclear import factor importin alphas (VvImpα and VvImpα4), localizes to the nucleus and triggers cell death when transiently expressed in tobacco (*Nicotiana benthamiana*) cells. Deletion of a nuclear localization signal (NLS) sequence from PvAVH53 or addition of a nuclear export signal (NES) sequence disrupted the nuclear localization of PvAVH53 and attenuated its ability to trigger cell death. Suppression of two tobacco importin-α genes, namely, NbImp-α1 and NbImp-α2, by virus-induced gene silencing (VIGS) also disrupted the nuclear localization and ability of PvAVH53 to induce cell death. Likewise, we transiently silenced the expression of VvImpα/α4 in grape through CRISPR/Cas13a, which has been reported to target RNA in vivo. Finally, we found that attenuating the expression of the Importin-αs genes resulted in increased susceptibility to the oomycete pathogen *Phytophthora capsici* in *N. benthamiana* and *P. viticola* in *V. vinifera*. Our results demonstrate that importin-αs are required for the nuclear localization and function of PvAVH53 and are essential for host innate immunity. The findings provide insight into the functions of importin-αs in grapevine against downy mildew.

## Introduction

Pathogenic oomycetes are responsible for some of the most widespread and devastating diseases of crop and ornamental plants. These diseases include potato late blight, caused by *Phytophthora infestans*, and grapevine downy mildew, caused by *Plasmopara viticola*. Similar to many other plant pathogens, oomycetes secrete a troop of effector molecules inside host cells to promote pathogenicity^1–4^. Many effectors, including RxLR proteins, have been shown to localize to the host cell nucleus and suppress the expression of genes that promote innate immune responses^5,6^. For example, a majority of the RxLR effectors studied from the oomycete pathogen *Hyaloperonospora arabidopsidis* target the plant cell nucleus^6^. The RxLR effectors Pi04089 and Pi04314 from *P. infestans* localize to the potato host cell nucleus; Pi04089 targets the K-homology (KH) RNA-binding protein (StKRBP1) to promote colonization, while Pi04314 enhances colonization by attenuating the induction of jasmonic acid- and salicylic acid-related immune responses^7,8^. Another *P. infestans* RxLR effector, PITG_22798, is transferred to the nucleus to induce cell death in tobacco^9^. In addition, activation of the innate immune response by the resistance protein R1 requires colocalization of the RxLR effector AVR1^10^. Although these studies underscore the importance of the nuclear localization of effector proteins in modulating plant defense, there have been few insights into the associated mechanism(s).

The nuclear envelope is highly selective for the dynamic exchange of macromolecules between the nucleus and cytoplasm^11–14^. The classic nuclear import pathway for proteins depends on the binding of a short nuclear localization signal (NLS) sequence by importin-α, interaction with importin-â, and shuttling of the protein through the nuclear pore complex^15,16^. Importin-α proteins typically contain three conserved domains: an amino-terminal importin-â-binding domain (IBB), tandem armadillo (Arm) repeats that interact with the NLS region of the cargo protein, and a carboxyl-terminal region that binds a nuclear export factor to facilitate its return to the cytoplasm^17,18^. Canonical NLSs are classified as monopartite NLSs, with a single cluster of basic amino residues (K[K/R]X] K/R]), and bipartite NLSs, containing two clusters ([K/R] [K/R]X_10-12_] [K/R]_3/5_) bridged by linker amino acids^19,20^.

Increasing evidence suggests that the nuclear localization of RxLR effectors is dependent on NLS-importin pathways. For example, the ability of CRN8 and CRN108 from *P. infestans* and PvRxLR16 from *P. viticola* to induce plant cell death is dependent on their nuclear localization^21–23^. The nuclear-targeted effector HaRxL106 from *H. arabidopsidis* was reported to interact with importin-α^24^. In *Nicotiana benthamiana*, silencing of the importin-α genes NbIMPα1 and NbIMPα2 via virus-induced gene silencing (VIGS) disrupted nuclear localization of the *P. infestans* effectors Nuk6 and Nuk7^12^ as well as the SAP11 effector from the phytoplasma *Candidatus* Phytoplasma asteris^25^. Furthermore, an engineered SAP11 protein in which the NLS was disrupted and a nuclear exclusion sequence was appended to the carboxyl terminus failed to target the nucleus and was associated with weakened pathogenicity^26^.

Several recent studies have also suggested that nuclear localization of pathogen effectors is required for triggering the plant innate immune response. For example, in Arabidopsis, the leucine-rich-repeat (LRR) innate immune receptor TN13 physically interacts with the importin-α *MOS6/Imp-*α*3*, and *mos6* mutants showed enhanced susceptibility to the oomycete *Hyaloperonospora parasitica*^27,28^. The RxLR effector PvAvh74 from *P. viticola* also requires nuclear localization to induce immune responses in tobacco^29^. Thus, interactions between importin-α and both effectors and innate immunity-related proteins play important roles in plant defense responses against pathogens^12,27,30–32^.

In previous preliminary work, we observed that the *P. viticola* effector PvAVH53 localized to the nucleus and triggered cell death in *N. benthamiana*^33^. In this study, we report that PvAVH53 physically interacts with importin-αs (VvImpα/VvImpα4, and NbImpα1/NbImpα2) from *V. vinifer*a and *N. benthamiana* and that these interactions are crucial for the function of the effector PvAVH53. Engineered PvAVH53 proteins lacking the NLS or containing a nuclear export signal (NES) localized to both the nucleus and cytoplasm of *N. benthamiana* cells and showed significantly attenuated activity to trigger cell death. In addition, both the nuclear localization and cell death activity of PvAVH53 were repressed in tobacco cells in which the expression of two VvImpα4 homologs, namely, NbImpα1 and NbImpα2, was repressed. Likewise, we transiently silenced the expression of VvImpα/α4 in grape through CRISPR/Cas13a, which has been reported to target RNA in vivo. Finally, attenuating the expression of the Importin-αs genes resulted in increased susceptibility to the oomycete pathogen *Phytophthora capsici* in *N. benthamiana* and *P. viticola* in *V. vinifera*. Taken together, these investigations demonstrate that importin-α is required for the nuclear localization and function of PvAVH53. They provide a solid foundation for advanced study of importin-αs in grapevine against downy mildew.

## Results

### Nuclear localization of PvAVH53 is essential for triggering cell death

**Fig. 1:**
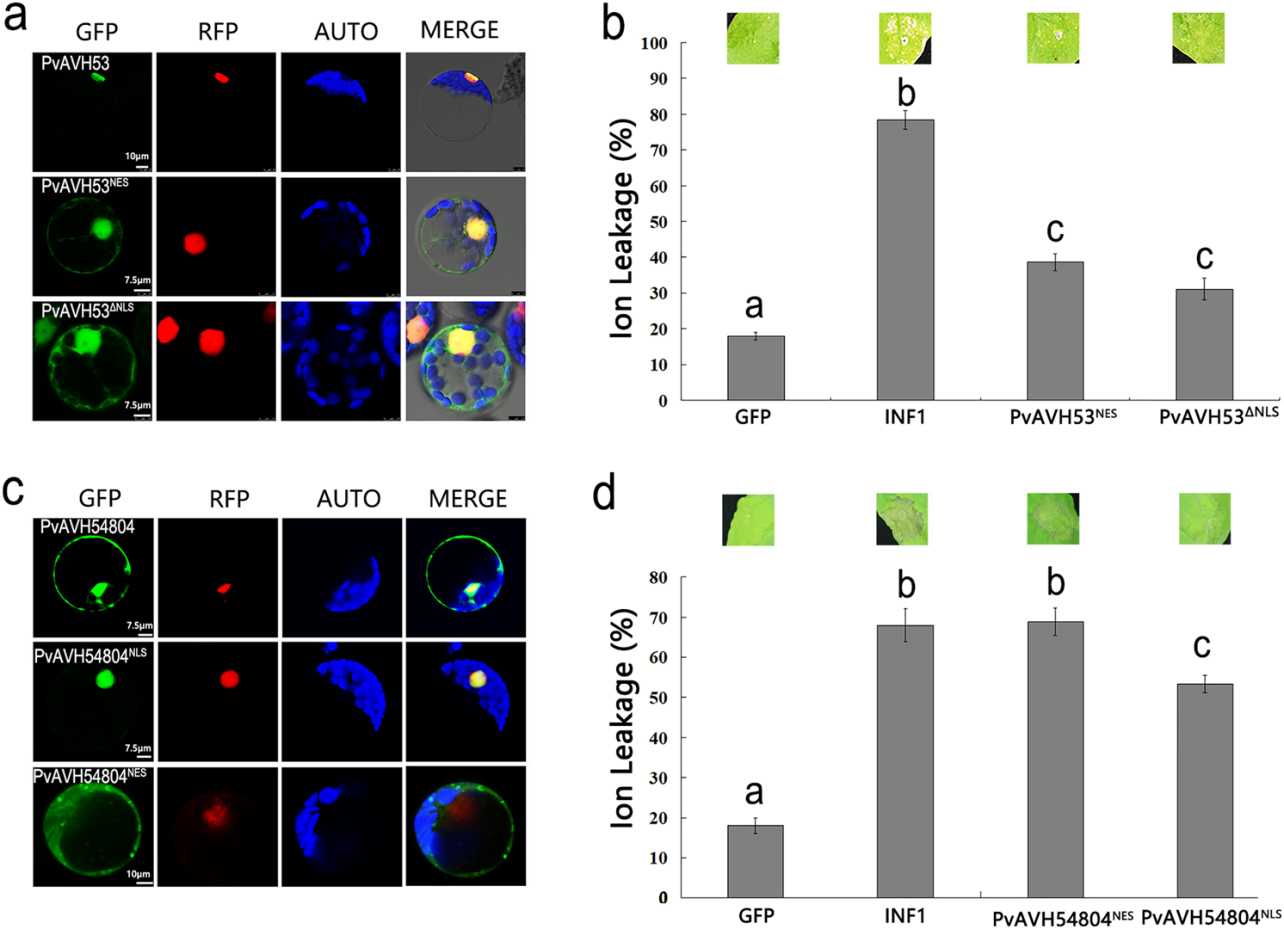
PvAVH53 requires nuclear localization to trigger cell death but PvAVH54804 does not. **a** Transient expression of PvAVH53 with a nuclear export signal (PvAVH53^NES^) or nuclear localization signal (NLS) deletion (PvAVH53^ΔNLS^) cotransformed with an NLS-mCherry marker in *Nicotiana benthamiana* protoplasts, showing that both mutants of PvAVH53 failed to stabilize nuclear localization. **b** Transient expression of the PvAVH53 mutants in *N. benthamiana* leaves by agroinfiltration showed that PvAVH53 mutants failed to trigger cell death. **c** Transient expression of PvAVH54804 with a nuclear export signal (NES) or nuclear localization signal (NLS) cotransformed with an NLS-mCherry marker in *N. benthamiana* protoplasts. **d** Transient expression of the PvAVH54804 mutants in *N. benthamiana* leaves by agroinfiltration showed that PvAVH54804^NLS^ failed to trigger cell death. Photos were captured at 6 d post infiltration. Ion leakage from the infiltrated leaf discs was measured as a percentage of leakage from boiled discs to quantify cell death. Scale bar = 5–10 μm. The data are the means ± SEs based on three independent replicates (Student’s *t*-test, *P* < 0.01)

Previously, we identified several *P. viticola* RxLR effectors capable of triggering cell death when expressed transiently in *N. benthamiana* cells. Our preliminary results suggested that among these effectors, PvAVH53 and PvAVH54804 induced cell death the most rapidly and at the lowest apparent concentration^33^. Imaging of *N. benthamiana* protoplasts transiently expressing PvAVH53-GFP or PvAVH54804-GFP showed that PvAVH53-GFP localizes to the nucleus, whereas PvAVH54804-GFP accumulates in both the nucleus and cytoplasm (Fig. 1a, c). To determine whether nuclear localization is required for PvAVH53-mediated induction of cell death, a NES sequence was appended to the carboxyl terminus of PvAVH53 (PvAVH53^NES^), or the NLS peptide sequence was deleted from PvAVH53 (PvAVH53^ΔNLS^), and these modified PvAVH53s were expressed in tobacco leaf protoplasts. In both cases, a substantial increase in fluorescence was observed outside the nucleus, indicating substantial interference with nuclear accumulation of PvAVH53 (Fig. 1a). We observed similar results when expression was evaluated in *V. vinifera* protoplasts (Fig. S1a). We also modified PvAVH54804 by the addition of a C-terminal NES or NLS peptide (PvAVH54804^NES^, PvAVH54804^NLS^, respectively). In contrast to the modified PvAVH53, the PvAVH54804^NES^ and PvAVH54804^NLS^ proteins showed essentially complete targeting to the nucleus or cytoplasm in *V. vinifera* and *N. benthamiana* leaf cells (Fig. S1b; Fig. 1b). To determine whether a change in subcellular localization affected the function of the effectors, the effector-GFP fusion proteins were transiently expressed in *N. benthamiana* leaf cells using agroinfiltration, and ion leakage was measured 4 d after infiltration^34^. We found that PvAVH53­^NES^ showed a strongly reduced ability to induce cell death compared with nonmodified PvAVH53 (Fig. 1b). We also found that PvAVH54804^NES^ triggered cell death to a similar degree as nonmodified PvAVH54804, while PvAVH54804^NLS^ showed a significantly decreased ability to induce cell death (Fig. 1d). Taken together, these findings demonstrated that nuclear localization of PvAVH53 is required for full activity to induce cell death, whereas PvAVH54804 mostly requires cytoplasmic localization to induce plant cell death.

### PvAVH53 interacts with the importin VvIMPα/α4

**Fig. 2:**
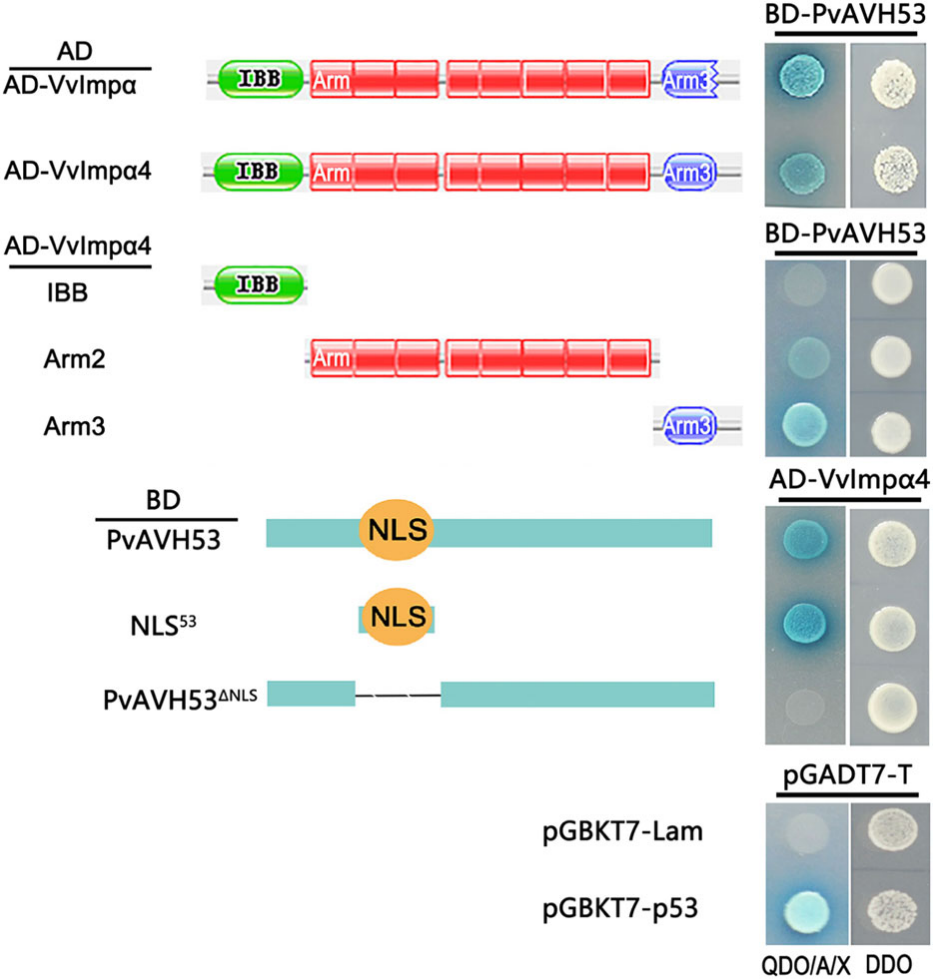
Y2H assay showing that PvAVH53 interacts with *Vitisvinifera* VvImpα/α4. The short segment of PvAVH53 containing the NLS (124–134 amino acids) interacts with the two arm domains of VvImp α4, as evidenced by growth on DDO (minimal medium, double dropout: SDLeu/-Trp) and QDO/A/X (minimal medium, quadruple dropout: SD-Ade/-His/-Leu/-Trp) in the presence of Aba and Xα-Gal. Left panel: Schematic diagram of the full length and deletion constructs of VvImpα4 and PvAVH53. Right panel: Confirmation of the interaction by cotransformation of different plasmids

We previously found that PvAVH53 localizes to the nucleus when expressed transiently in *N. benthamiana* cells^33^. To identify potential interaction partners of PvAVH53 in *V. vinifera*, we conducted a yeast two-hybrid assay using PvAVH53 as bait in combination with a *V. vinifera* cDNA expression library. This resulted in the identification of multiple clones representing two distinct genes, both previously annotated as importin subunit alpha, namely, VvImpα and VvImpα4, through an NCBI BLAST search. To evaluate the interaction between PvAVH53 and VvImpα/α4, we cloned a cDNA from *V. vinifera* encoding the full-length, 1,608-bp *VvIMPα4* and 1590-bp *VvIMPα* open reading frames (ORFs) into the prey vector and a cDNA encoding PvAVH53 into the bait vector. This Y2H assay showed that the two proteins interact in yeast cells (Fig. 2). VvImpα4 contains an amino-terminal importin beta-binding (IBB) domain (amino acids 100–200) and nine armadillo (Arm)/beta-catenin-like repeats (Fig. 2). A short, amino-terminal segment of the VvImpα4 protein containing the IBB domain showed no interaction with PvAVH53 (Fig. 2). An internal segment of VvImpα4 lacking both the N-terminus and C-terminus and expressing only eight of the nine Arm domains showed a weak interaction with PvAVH53 (Fig. 2). Interestingly, a short segment of VvImpα4 containing only the C-terminal Arm domain showed a strong interaction with PvAVH53 (Fig. 2).

**Fig. 3:**
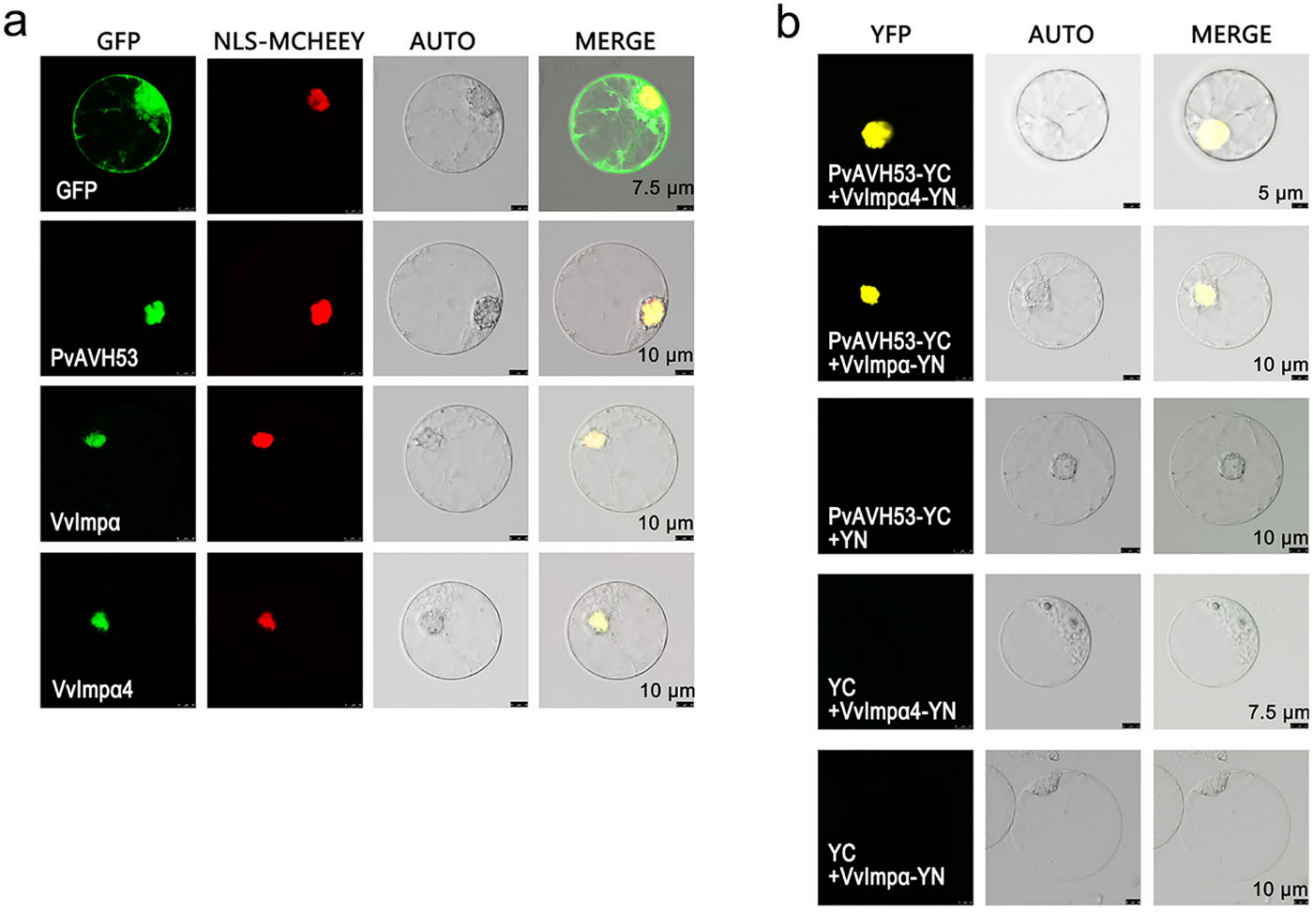
PvAVH53 and VvImpαs localize to and interact in the *Vitis vinifera* cell nucleus. **a** Subcellular localization of *V. vinifera* nuclear import factors VvImpα4/VvImpα, PvAVH53, and GFP as controls cotransformed with NLS-mCherry (as the nuclear localization marker) in *V. vinifera* protoplasts. **b** A BiFC assay confirmed that VvImpαs interacted with PvAVH53. Photos were taken after protoplasts were incubated for 20–24 h under weak lighting at 25 °C. Scale bar = 5–10 μm

We also engineered deletion mutants of PvAVH53 to identify specific regions of the PvAVH53 protein important for interaction with VvImpα4. We found that a short segment of PvAVH53 containing the NLS was both required and sufficient for interaction with VvImpα4 (Fig. 2). We also used this two-hybrid assay to evaluate the interaction between PvAVH53 and VvImpα. Similar to VvImpα4, a strong interaction was observed when a full-length VvImp-α clone was used as bait (Fig. 3a). Finally, we used the yeast two-hybrid assay to evaluate the interaction between VvImpa4 and a panel of 10 *P. viticola* RxLR-type effectors^33^ previously identified in our laboratory (the details of the effectors are provided in the supplementary material in Table S1). With two exceptions (PvAVH1 and PvAVH54804), all effectors showed interactions with VvImpα4 (Fig. S2). These results suggest that grapevine importin-α proteins may participate in the nuclear import of a wide range of RxLR effectors.

### PvAVH53 and VvImpαs localize to and interact in the nucleus

To further document the interaction between PvAVH53 and VvImpαs, we transformed *V. vinifera* protoplasts with clones expressing the proteins fused with GFP and examined their intracellular localization via fluorescence microscopy. An NLS-mCherry marker was coexpressed to visualize the nucleus. This revealed that both PvAVH53-GFP and VvImpα-GFP fusion proteins localized to the nucleus (Fig. 3a). We further analyzed the interaction using bimolecular fluorescence complementation (BiFC). PvAVH53 carrying a carboxyl-terminal fragment of YFP (PvAVH53-YC) was coexpressed with VvImpα or VvImpα4 with amino-terminal fusion of YFP (VvImpα-YN or VvImpα4-YN, respectively) in *V. vinifera* protoplasts. YFP fluorescence was observed in the nucleus, indicating that VvImpα and VvImpα4 interact with PvAVH53 in the host nucleus (Fig. 3b).

In summary, these two complementary approaches provided solid evidence that the RxLR effector PvAVH53 interacts with *V. vinifera* VvImpα and VvImpα4.

### PvAVH53 interacts with Importin-αs in tobacco leaf cells

**Fig. 4:**
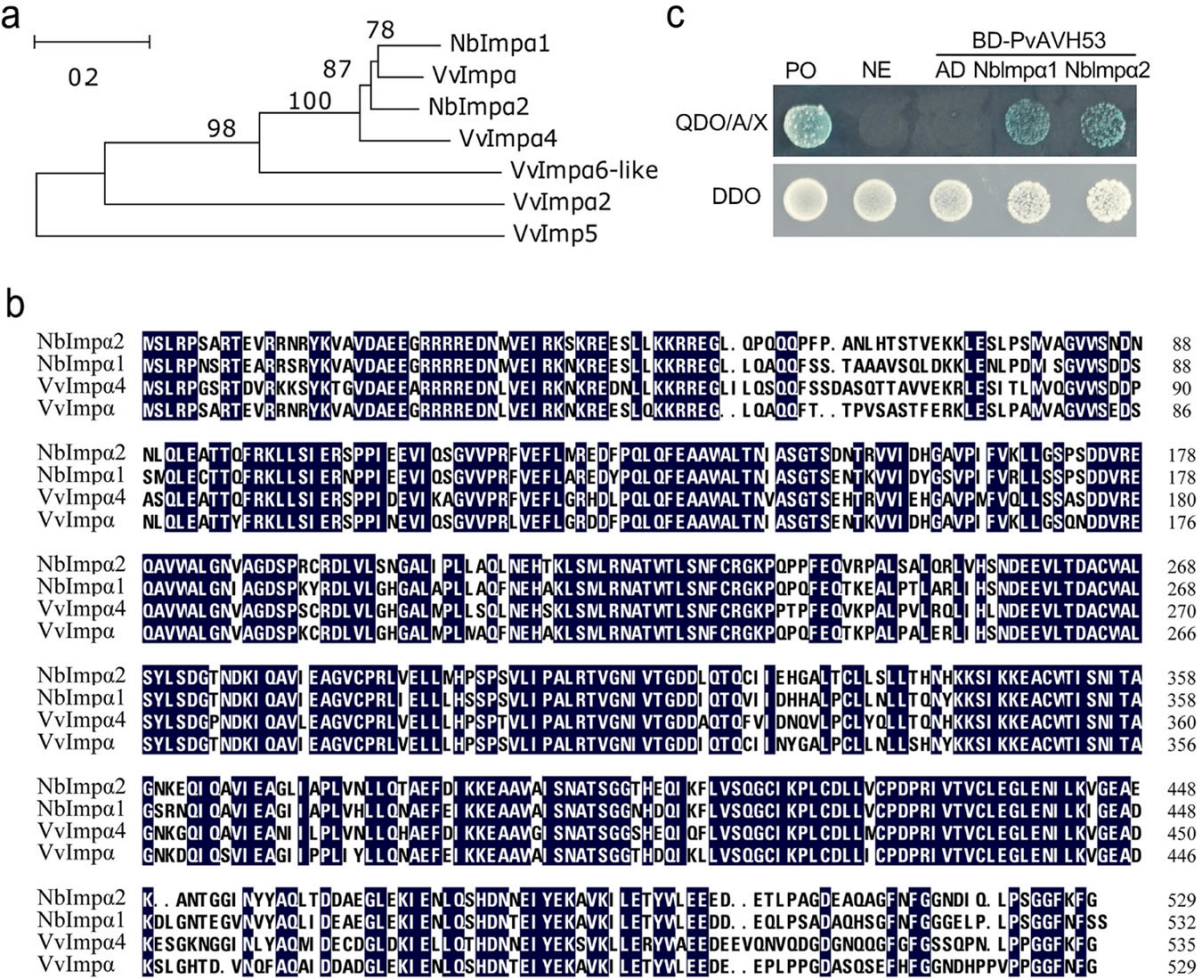
PvAVH53 interacts with *Nicotiana benthamiana* NbImpαs. **a** Phylogenetic analyses of the VvImpαs (VvImpα XP_002274422.1, VvImpα2 XP_002282816.1, VvImpα4 XP_002281670.1, VvImpα6-like XP_010646172.1, VvImpα5 XP_002281591.1), and NbImpαs (NbImpα1: EF137253.1 and NbImpα2: EF137254.1) were conducted with MEGA5 software. **b** Sequence analysis of the VvImpα/α4 and NbImpα1/2. The sequence alignment of the NbImpαs and VvImpαs indicated that VvImpα and VvImpα4 showed high sequence identity with the two NbImpαs. **c** NbImpα1/2 interact with PvAVH53 in yeast Y2H Gold cells. The yeast cells grew and turned blue on DDO (SD-Leu/-Trp) and QDO/A/X (SD-Ade/-His/-Leu/-Trp) in the presence of Aba and X-α-Gal

VvImpα and VvImpα4 show strong amino acid sequence homology (up to 86.3%) and close phylogenetic relationships with two importin-αs from *N. benthamiana*, designated NbImpα1 and NbImpα2 (Fig. 4). To evaluate the interaction between PvAVH53 and these two importin-αs from tobacco by the yeast two-hybrid approach, we cloned cDNAs from *N. benthamiana* encoding the full-length ORFs of NbImpα1 and NbImpα2 into the bait vector and PvAVH53 cDNA into the prey vector. We also used BiFC to evaluate interactions between PvAVH53 and tobacco importin-αs. PvAVH53-YC was coexpressed with NbImpα1-YN or NbImpα2-YN in *N. benthamiana* protoplasts. YFP fluorescence was observed in the nucleus, indicating that NbImpα1 and NbImpα2 interact with PvAVH53 in the nucleus (Fig. S3).

### Silencing of NbImpα1/2 reduces plant growth and disturbs the nuclear localization of PvAVH53

**Fig. 5:**
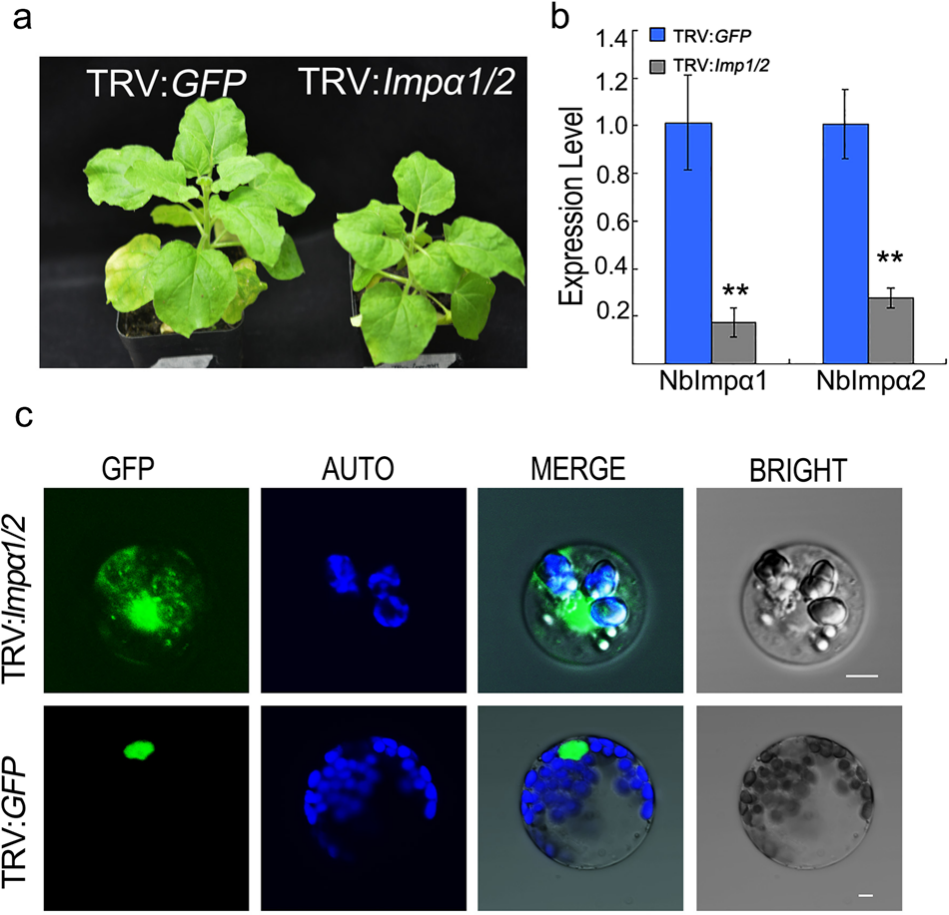
NbImpα1/2 silencing reduces plant growth and disturbs the nuclear localization of PvAVH53. **a** Morphology of *N. benthamiana* plants with TRV:*GFP* (control) and TRV:*Impα1/2*. **b** Relative quantification of the expression of NbImpα1/2 with TRV constructs using qRT-PCR. The NbEF1α gene was used as an internal control. (**c**) Subcellular localization in leaves of control (TRV:GFP) and Impα1/2-silenced plants analyzed by confocal microscopy. Photos were taken after protoplasts were incubated for 20–24 h under weak lighting at 25 °C. Scale bar = 10 μm. The experiments were repeated three times with similar results, and at least two protoplasts were observed each time

PvAVH53, but not PvAVH54804, contains a predicted NLS at amino acids 124–134 (AA). Importin-αs mediate the nuclear import of various cargo proteins carrying an NLS signal. To determine whether importin-αs are involved in the cell death-triggering activity of PvAVH53, we silenced both NbImpα1 and NbImpα2 expression in *N. benthamiana* using VIGS^2^. The primers were designed to amplify an ~0.6 kb fragment of NbImpα1 and NbImpα2, as described in Schornack et al 2010^21^, which was then ligated into TRV2^35^. *N. benthamiana* leaves were then subjected to agroinfiltration with TRV1 and a mixture of TRV2:*NbImp1&NbImpα2* and TRV2:*GFP* (TRV2:*GFP* was included as a negative control to visualize TRV infection). Primers for detection were designed for specificity with the silenced segment of NbImpα1/2. Leaves of the silenced *N. benthamiana* plants grew more slowly than those of the TRV:*GFP* control (Fig. 5a), and RT-PCR results revealed that NbImpαs transcript levels in the leaves of silenced plants were significantly lower than those in the control leaves (Fig. 5b). We also isolated protoplasts from NbImpα1/2-silenced plants and introduced the GFP:PvAVH53 construct. Confocal microscopy showed that the subcellular localization of PvAVH53 in NbImpα1/2-silenced protoplasts was similar to that of PvAVH53^NES^ in noninfiltrated tobacco leaf cells (Fig. 5c), indicating that NbImpα1/2 was successfully silenced in *N. benthamiana* and that PvAVH53 was relocated in the plant nucleus and cytoplasm.

### Silencing of NbImpα1/2 diminishes cell death triggered by PvAVH53 and promotes the susceptibility of *N. benthamiana* to *Phytophthora capsici*

**Fig. 6:**
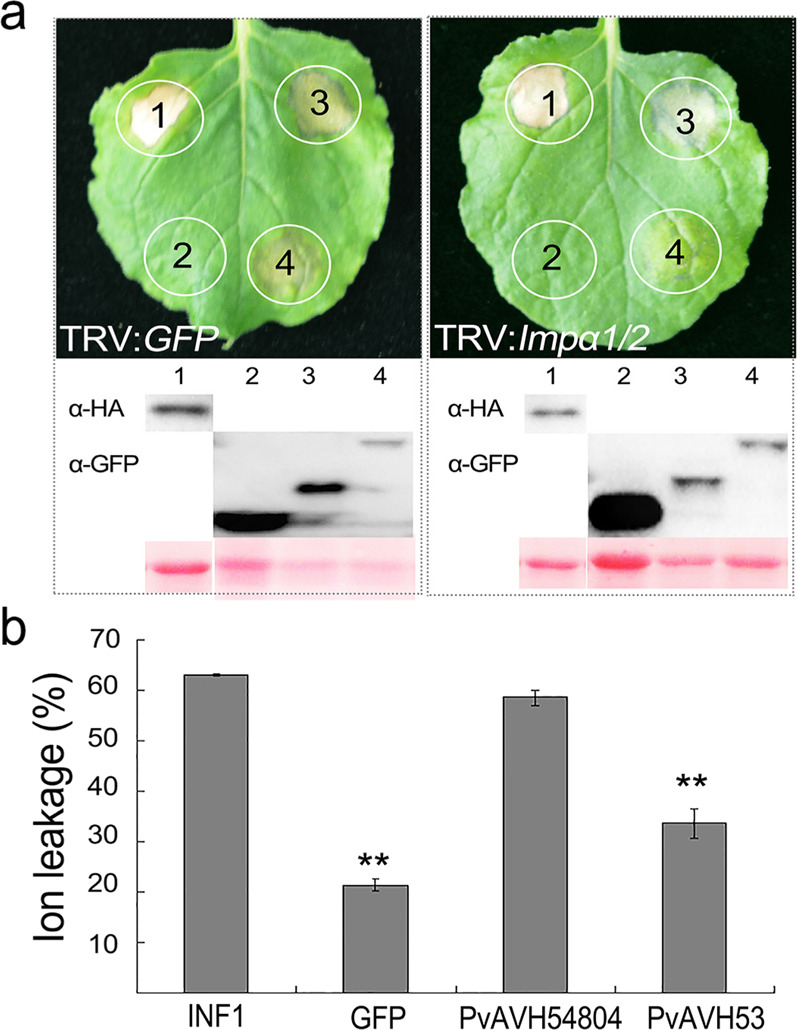
NbImpα1/2 silencing decreases the cell death triggered by PvAVH53 but PvAVH54804 does not. **a** Leaves of control (TRV:*GFP*) and Impα1/2-silenced plants were agroinfiltrated with PvAVH53/PvAVH54804 expression plasmids. Proteins were extracted from infiltrated spots to analyze the expression (spot number: 1, INF1; 2, GFP; 3, PvAVH54804; 4, PvAVH53). **b** Ion leakage (%) measurement at infiltration sites. The data are the means ± SEs based on three independent replicates (Student’s *t*-test: **P < 0.01)

Detection of the ion leakage of the control (TRV-GFP and WT) and NbImpα1/2-silenced tobacco leaves showed that virus-induced gene silencing (VIGS) in *Nicotiana benthamiana* had no significant effect on ion leakage (Fig. S4). Both PvAVH53 and PvAVH54804 were transiently expressed by agroinfiltration in NbImpα1/2-silenced and control *N. benthamiana* leaves. PvAVH54804 induced cell death in both NbImpα1/2-silenced and control leaves. In contrast, PvAVH53 showed only a weak ability to induce cell death and to promote ion leakage in Impα1/2-silenced leaves compared with the control (Fig. 6a, b). Based on this finding, we concluded that the nuclear localization of PvAVH53, as well as its ability to trigger cell death, depends on NbImpα1/2.

To study the role of NbImpα1/2 in the defense against pathogens, we evaluated the potential of *P. capsici* to infect leaves from NbImpα1/2-silenced tobacco plants. We found that the lesions on NbImpα1/2-silenced tobacco plants were larger than the lesions on control plants (Fig. S5a). Measurement of the lesion area of *P. capsici* provided independent evidence that NbImpα1/2-silenced tobacco plants were more susceptible to *P. capsici* (Fig. S5b).

### Silencing of VvImpα/α4 disorders the sublocalization of PvAVH53 and promotes the susceptibility of *V. vinifera* to *P. viticola*

**Fig. 7:**
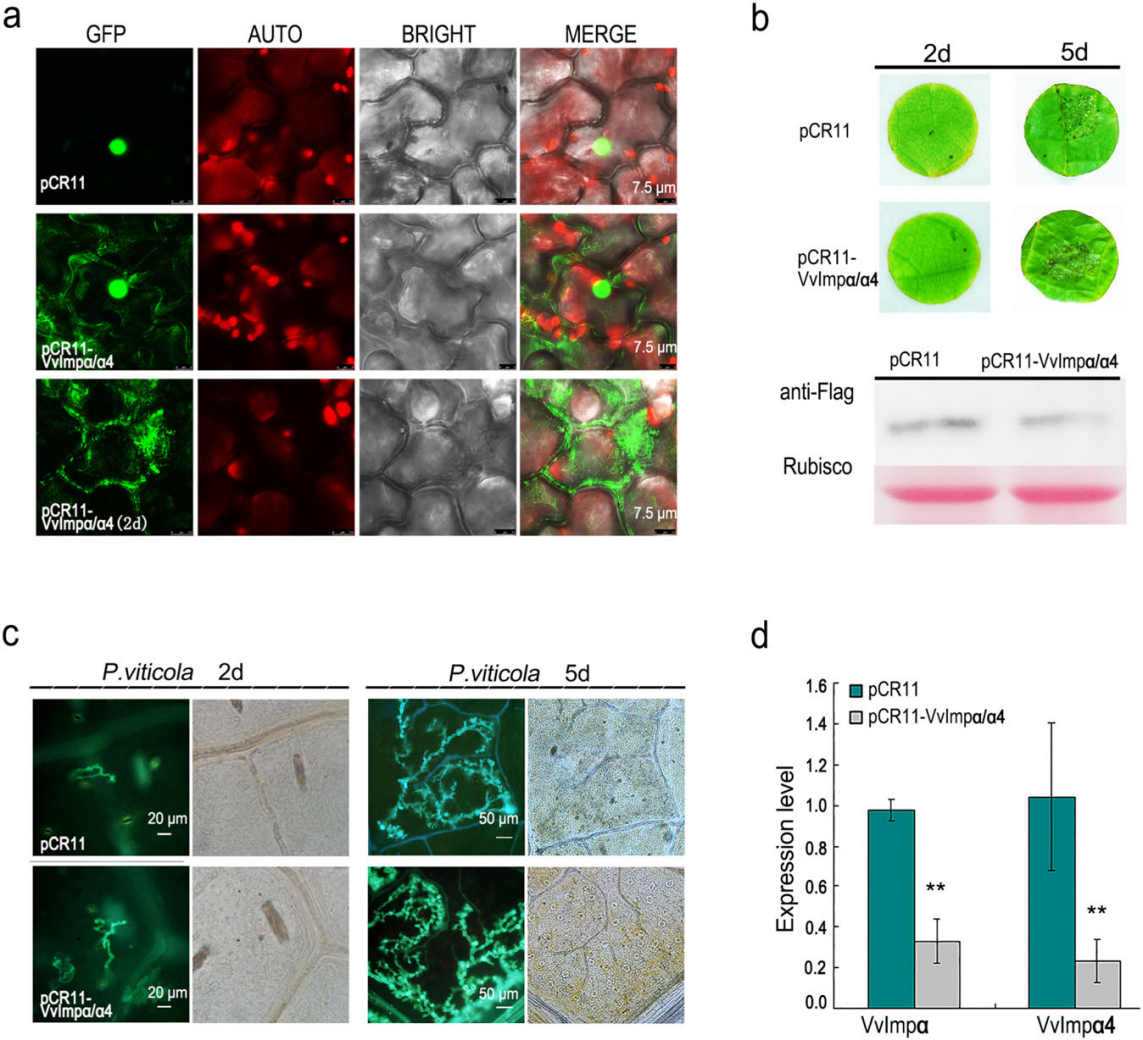
*VvImpα/α4* silencing changes the sublocalization of PvAVH53 and promotes the susceptibility of *V. vinifera* to *P. viticola*. **a** Subcellular localization of PvAVH53 on leaves of control (pCR11) and pCR11-*VvImpα/α4-*silenced grape leaves was analyzed by confocal microscopy. Scale bar = 5–7.5 μm. **b** Phenotype of control (pCR11) and pCR11-*VvImpα/α4-*silenced grape leaves infected with *P. viticola*. Leaf discs were photographed at 2 days and 5 days post infection. Western blotting detected Cas13a protein expression in control and *VvImpα/α4-*silenced grape leaves. **c** *P. viticola* development at inoculation sites in leaf discs of control and *VvImpα/α4-*silenced grape leaves was revealed by aniline blue staining. **d** Relative quantification of the expression of VvImpα/α4 in control and *VvImpα/α4-*silenced grape leaves via qRT-PCR. The VvActin (AY680701) gene was used as an internal reference. The data are the means ± SEs based on three independent replicates (Student’s *t*-test: **P < 0.01)

To demonstrate the roles of VvImp αs in the nuclear localization of PvAVH53 in grape and in the defense against *P. viticola*, we transiently silenced the expression of VvImpα/α4 in grape through CRISPR/Cas13a, which has been reported to target RNA in vivo^36,37^. Transient expression of PvAVH53 in grape leaves cotransformed with pCR11 or pCR11-*VvImpα/α4* showed that PvAVH53 also failed to target the nucleus only in the host plant. Even though PvAVH53 was expressed in grape after 2 days of infection with pCR11-*VvImpα/α4*, its localization was obviously rearranged in the cytoplasm (Fig. 7a). Then, we evaluated the potential of *P. viticola* to infect leaves from control (pCR11) and pCR11-*VvImpα/α4*-silenced grape plants. Leaf discs obtained from the grape leaves were agroinfiltrated with control (pCR11) and pCR11-*VvImpα/α4* expression plasmids, infected with a *P. viticola* sporangium suspension, and cultured in a greenhouse under a 16-h light/8-h dark photoperiod at 25 °C (light) and 18 °C (dark). We found that sporangia on *VvImpα/α4*-silenced grape leaves were more abundant than sporangia on control grape leaves (Fig. 7b). Western blotting showed that the Cas13a protein was expressed in control and *VvImpα4-*silenced grape leaves, and quantitative real-time PCR revealed that VvImpα and VvImpα4 transcript levels in *VvImpα/α4*-silenced leaves were significantly lower than those in the control leaves (Fig. 7b, d). To assess *P. viticola* development in control and *VvImpα/α4-*silenced grape leaves, the infection sites were stained with aniline blue and observed by a fluorescence microscope at 2 days and 5 days post infection, respectively. Observation of infection at 2 days showed that the encysting zoospore formed a germinative tube in the control and VvImp*α4*-silenced grape leaves. At 5 days post infection, hyphae were obviously visible in the leaf tissues of the control and VvImp*α4*-silenced grape plants, but the hyphae were more abundant and sporangiophores or sporangia were apparent in *VvImpα/α4*-silenced grape leaves (Fig. 7b, c).

## Discussion

The success of a pathogen depends on its capacity to overcome the host plant’s innate immune responses. Effectors play a significant role in the plant-pathogen battle by interrupting host cellular processes and promoting colonization. The proximity of the plant cell nucleus to the developing oomycete haustorium has been observed more than once, suggesting that the haustorium may influence the intracellular architecture of the cell for efficient delivery of effectors to disturb nuclear defensive responses^6,38,39^. In this study, we found that the RxLR-type effector PvAVH53 from *P. viticola* interacts with VvImpα/α4 and enters the plant nucleus through the classic nuclear import pathway and triggers cell death. To our knowledge, this is the first evidence that *P. viticola* effectors share the canonical nuclear import machinery by targeting the adaptor importin-α, as has been shown in other oomycetes, such as *Hyaloperonospora arabidopsidis* and *P. infestans*.

Previous experiments have indicated that the *P. viticola* effectors PvAVH53 and PvAVH54804 can induce cell death in *N. benthamiana* and are localized strictly in the nucleus or both in the nucleus and cytoplasm, respectively^33^. Our experiments further showed that PvAVH53 and PvAVH54804 induce cell death in *N. benthamiana*. Many studies, especially in the oomycete pathogen model *P. infestans*, have investigated interactions between pathogens and plant host cells^4,40^, but the host targets of the *P. viticola* effectors and associated pathogenic mechanisms have rarely been described.

In our experiments, the addition of a nuclear export signal (NES) sequence to PvAVH53 or removal of the NLS peptides from PvAVH53 did not completely alter the subcellular localization; rather, these PvAVH53 mutants changed from being localized in the nucleus to being distributed in the nucleus and cytoplasm in both *V. vinifera* and *N. benthamiana*. However, PvAVH54804, with the same NES signal, was completely localized in the cytoplasm or distributed in only the nucleus with the NLS signal, demonstrating that the transport signals (namely, NLS and NES) were functional for protein transport; thus, PvAVH53 may have evolved other features that contribute to strong targeting to the plant cell nucleus, perhaps by interacting with endogenous host proteins. Moreover, all the modified effectors showed a significantly decreased ability to trigger cell death, indicating that the differences in localization may reflect the functional differences of the proteins, or to function properly based on the localization of the effectors. We identified VvImpαs as targets of PvAVH53 using a yeast two-hybrid assay. As reported in previous studies, importin-αs exhibit three highly conserved structural domains: the importin-â-binding domain, ten armadillo (Arm) repeats for the binding of NLS-carrying proteins, and a C-terminal region that functions as a nuclear export factor binding site^41–43^. VvImpα4 contains nine tandem Arm motifs, including a C-terminal typical Arm motif, rather than ten arm repeats as previously reported^44^. Consistent with previous studies, we found that PvAVH53 interacts with Arm repeats of VvImpα4, especially the C-terminal Arm, via its NLS sequence. Previous studies have revealed that transport factors are targeted by pathogen effectors; for example, the *P. infestans* RXLR effector AVR1 targets the exocyst subunit Sec5 to disturb vesicle trafficking, potentially regulating plant immunity^45^. HaRxL106 from _­_*Hyaloperonospora arabidopsidis* interacts with the importin-α Arm repeat domain via an NLS^24^. In rice, the bacterial pathogens *Xanthomonas oryzae* pv*. oryzae* (Xoo) and *Xanthomonas oryzae* pv*. oryzicola* (Xoc) target OsImpα1a and OsImpα1b to facilitate infection^46^. However, multiple imp-α homologs/isoforms exist in plants^12^, and these may associate with distinct groups of cargo proteins. The formation of cargo/importin-α complexes is not always unique for effectors with NLS sequences. For example, in a study of interactions between importin-α and 83 effectors from *Hpa* and *Pseudomonas syringae*, the *Hpa* effector HaRxL106 was found to target several importin-αs (including MOS6, importin-α1, importin-α2 and importin-α4), whereas the effector HaRxL445 interacted specifically with MOS6^24,47^. Our study addressed interactions between the VvImpα4 homologs VvImp-α in *V. vinifera* and PvAVH53. Furthermore, there were an additional 11 RxLR-type effectors; the nine that were localized in only the nucleus interacted with VvImpα4, but the two non-nuclear effectors failed to target VvImpα4. These results implied that effectors containing the NLS localized in the plant nucleus and interacted with VvImpα4, but effectors lacking the NLS did not. Moreover, these interactions were specific and general simultaneously for nuclear-only effectors, and effectors with non-NLS signals may also have evolved other features that allow them to target the plant cell cytoplasm and the nucleus at the same time.

Phylogenetic analyses showed high conservation and homology in VvImpαs and NbImpαs, indicating that the function of NbImpαs in *N. benthamiana* is likely to apply to VvImpαs in *V. vinifera*. Our experiments using Y2H and BiFC assays support the interaction between the orthologs NbImpα1/2 and PvAVH53. We used BiFC to show the interaction between PvAVH53 and Importinαs (VvImpα4, VvImpα, NbImpα1, and NbImpα2) in the plant cell nucleus. Moreover, we took advantage of the virus-induced gene silencing (VIGS) and CRISPR/Cas13a systems to knock down the expression of two VvImportin-α4 homologs from *N. benthamiana* (NbImpα1 and NbImpα2) and VvImpα/α4 from grape. The CRISPR/Cas13a system driven by suitable promoters for dicot and monocot plants was introduced to target RNA^37^. It was previously reported that the nuclear localization of *P. infestans* Nuk6 and Nuk7, but not Nuk12, was affected in NbImpα1/2-silenced tobacco^12^. Likewise, the PvAVH53 fluorescence-labelled protein was no longer localized in the nucleus in these NbImpα1/2-silenced tobacco and VvImpα4-silenced grape leaves, providing evidence that PvAVH53 relies on the importin α-dependent nuclear import pathway to stabilize nuclear localization. Furthermore, transient expression of PvAVH53 and PvAVH54804 in NbImpα1/2-silenced tobacco showed the opposite effects: PvAVH53 failed to induce cell death, but PvAVH54804 triggered cell death. Moreover, reducing NbImp-α1/2 expression levels also increased the susceptibility to *P. capsici*, suggesting that NbImp-α1/2 may be directly or indirectly involved in transporting PvAVH53 to the nucleus and is required for immunity to *P. capsici*. At the same time, we used the CRISPR/Cas13a system to silence VvImpα/α4 in grape and decrease the resistance to *P. viticola*. The results showed that VvImpαs was required for the nuclear localization of PvAVH53 and was also required for overcoming *P. viticola* infection in grape. These results are consistent with a previous study showing that mutation of MOS6 (AtImportin-α3) promoted susceptibility to the oomycete plant-pathogen *Hyaloperonospora parasitica*^27^. As previously reported, Importin-αs benefit infection by both viruses and bacteria, but the opposite is true for oomycetes. All the studies suggested that in nuclear transport trafficking, which is central to plant-pathogen interactions, Importinα has multiple function in plants and plays a significant role in plant defense and development. A previous study found that some proteins from pathogens are dependent on NbImp-α1/2 or close homologs for nuclear import, but some can target the nucleus independently of α-Importins^12^. Altogether, these results demonstrate that VvImpα4 or similar α-Importins (such as NbImp-α1/2) are required for PvAVH53 to target the nucleus and induce cell death in *N. benthamiana* and increase susceptibility to *P. capsici* and *P. viticola*. However, importinαs, for plant immunity, are a double-edged sword. On the one hand, they transport plant immune response-related proteins such as the late blight resistance protein R1, the shortened NLR protein TN13, and the polypolymerase PARP2 to contribute to immunity^10,28,48^. On the other hand, importin-αs also benefit pathogens, such as *Pelargonium* line pattern virus and *Xanthomonas oryzae* pv*. oryzicola*^9,10,49^.

In summary, we conclude that PvAVH53 depends on Importin-αs for transport to the nucleus in *V. vinifera* and *N. benthamiana* and that the canonical importin-α import pathway contributes to plant immunity or the pathogenicity of plant pathogens. To date, our understanding of how *P. viticola* effectors modulate host immunity remains limited. Future studies will focus on the targets of pathogen effectors in the host nucleus and the mechanism involved in the response to infection.

## Materials and methods

### Plasmid constructions

The sequence and accession numbers for effector proteins are listed in Table S1. Effector genes, minus signal peptide sequences, were cloned from *P. viticola* (strain YL) genomic DNA using PCR and oligonucleotide primers, as listed in Table S2. Incorporation of NES (nuclear export signal) and NLS (nuclear import signal) sequences into the cloned effector genes was previously reported by Du et al, 2015^19^. The modified effector gene sequence was ligated into a derivative of the pCAMBIA2300 plasmid vector containing the green fluorescent protein (GFP) sequence. The oligonucleotide primers used in PCR-based cloning of *IMPORTIN*-α homologs are listed in Table S2. For the gene silencing assay, a segment of NbImpα1/NbImpα2 was amplified from *N. benthamiana* cDNA using the primers Impα1/2-TRV2-F and Impα1/2-TRV2-R and ligated into the plasmid vector pTRV2^25^. For CRISPR/Cas13a vector construction^37^, the target sgRNA of *VvImpα* and *VvImpα4* was designed using online software (http://crispr.dfci.harvard.edu/SSC/). Next, the forward and reverse primers of sgRNA were mixed equally at 98 °C for 5 min and then placed in an ice bath for 5 min. Finally, the products were ligated into the vector pCR11 described by Zhang Tong et al (2019)^48^. The constructs were transformed into *Escherichia coli* strain Top10 and subsequently cultured at 37 °C on agar-solidified LB (Luria-Bertani) medium with screening antibiotics.

### Plant materials, fungal materials and inoculation

Leaves of 4- to 5-week-old *N. benthamiana* and *V. vinifera* cv. Thompson Seedless were prepared for the following experiments. *N. benthamiana* plants were grown in a greenhouse for use in both Agrobacterium-mediated transient gene expression assays and *P. capsici* infection assays. Inoculation of leaves with *P. capsici* and *P. viticola* was carried out as described previously^22,33^. Briefly, *P. capsici* cultures were maintained on 10% V8 juice agar medium at 25 °C in the dark. Sporulation was induced on 10% V8 juice mix medium (solid and liquid) by incubation at 4 °C. Zoospores were eluted in sterile water, and the concentration was adjusted to 1.0 × 10^4^ mL^−1^. Detached leaves were inoculated with a 10 μL droplet of zoospore suspension and incubated under high humidity at 25 °C in the dark. Lesion areas were measured 2 days after infection. One-centimeter diameter leaf discs of the susceptible *V. vinifera* cv. Thompson Seedless were inoculated with 20 μL of a *P. viticola* sporangial suspension (5 × 10^4^ sporangia/mL) for 1 day, and then, the remaining suspension was removed and placed on wet sterile filter paper in a growth chamber at 22 °C.

### Transient transformation expression assays

Plasmid constructs were introduced into *A. tumefaciens* strain GV3101 by electroporation, and transformants were selected on solidified LB medium containing kanamycin (50 μg/mL), gentamycin (50 μg/mL) and rifamycin (50 μg/mL). Recombinant Agrobacterium strains were grown in liquid LB medium at 28 °C with shaking at 200 rpm. After 20 h of growth, cells were collected by centrifugation at 5000 × g for 3 min, washed twice with 500 mM MgCl_2_, and then resuspended in infiltration medium (10 mM MgCl_2_, 10 mM MES (pH 5.7), 200 μM acetosyringone) to an OD_600_ of 0.4. Cells were then maintained for 3 h at 28 °C in the dark prior to infiltration^50^. Infiltration was performed with leaves of *N. benthamiana* and *V. vinifera* using a 1-mL syringe, and plants were further grown in controlled conditions under a photoperiod of 16-h light at 25 °C and 8-h dark at 18 °C. All experiments were performed at least three times.

### TRV-induced gene silencing

Analysis of gene function by virus-induced gene silencing (VIGS) was performed by using a tobacco rattle virus (TRV)-based vector^35,51^. VIGS constructs (pTRV2:GFP^52^ and pTRV2:NbImpα1/NbImpα2) were introduced into *A. tumefaciens* strain GV3101 by electrotransformation.

Bacterial cultures were grown to OD_600_ = 0.1 and then combined at a 1:1 ratio and injected into fully expanded leaves of 2-week-old *N. benthamiana* plants. Plants subjected to infiltration were grown for 2–3 weeks in a greenhouse under a daily regime of 16-h light at 22 °C and 8-h dark at 18 °C. Oligonucleotide primer sequences used for detecting the silenced gene by quantitative RT-PCR are given in Supplemental Table 2. The NbEF1α gene was used as an internal reference^29^.

### Bimolecular fluorescence complementation and confocal imaging

Analyses of intracellular protein interactions and colocalization using bimolecular fluorescence complementation (BiFC)^53^ assays were performed by using protoplasts derived from healthy and fully expanded tobacco leaves or callus tissue prepared from cv. Thompson Seedless. Protoplast preparation and polyethylene glycol (PEG)-mediated transformation of the protoplasts were carried out as previously described^54^. PvAVH53 and VvImpα gene fragments were cloned into pUC-SPYNE and pUC-SPYCE, respectively, to create PvAVH53-YN and VvImpαs-YC. The PvAVH53-YN and VvImpαs-YC constructs were cotransformed into protoplasts. Empty pUC-SPYNE and pUC-SPYCE vectors were used as negative controls. Protoplasts were incubated for 20–24 h under weak lighting at 25 °C prior to imaging by confocal microscopy (Germany, Leica TCS SP8). The wavelengths used for excitation of GFP, YFP and mCherry were 488, 630, and 561 nm, respectively.

### Protein extraction and western blotting

To detect the expression of recombinant proteins, protein extraction and western blotting were carried out as described in Chen et al. (2020)^33^. In brief, proteins extracted by using an extraction buffer were added to 1× loading buffer and incubated in a boiling water bath for 5 min. The supernatant was loaded on a 10% SDS-PAGE gel, and proteins were transferred to a PVDF membrane using a Trans-Blot cell (America, Biorad Trans-Blot SD). The membrane was incubated with 5% nonfat dry milk in TBST (20 mM Tris-HCl, 150 mM NaCl, 0.05% Tween-20) for 3 h at room temperature. Mouse anti-GFP monoclonal antibodies (ABclonal) were then added to the buffer at a ratio of 1:5000, and the membrane was shaken slowly overnight at 4 °C and then washed in TBST. Goat anti-mouse IRDye 800CW was then added at a ratio of 1:10,000, and the membrane was shaken for an additional 1 h. The membrane was washed five times in TBST for 5 min each time and then visualized using ChemiDocTM XRS + software.

### Bioinformatics and sequence analysis

NLS signal sequences were predicted using cNLS Mapper (http://nls-mapper.iab.keio.ac.jp/cgi-bin/NLS_Mapper_form.cgi#opennewwindow). The domain organization of VvImpα4 was analyzed using Pfam (http://pfam.xfam.org/).

Protein sequence alignment was performed by using DNAMAN BLAST. The phylogenetic tree was constructed using MEGA5 with the neighbor-joining method, 1000 replicates, and the pairwise deletion option.

### Visualization of cell survival by GUS staining

For evaluation of *N. benthamiana* cell survival following coinfiltration with pBI121::*GUS* and RxLR53 or RxLR54804, histochemical analysis of GUS activity was carried out 4 days after infiltration as described previously^55^. The GFP-effector fusion protein, GFP (as a negative control) and PGR107-NotI*::INF1* (as a positive control) were each coexpressed with PBI121::*GUS* in *N. benthamiana* leaves using agroinfiltration. Photographs were taken 4 d post infiltration.

### Trypan blue staining and aniline blue staining

For trypan blue staining, *N. benthamiana* leaves were immersed in trypan blue solution (10 g of phenol, 10 ml of glycerol, 10 ml of distilled water, and 0.02 g of trypan blue), boiled in a water bath for 2 min, incubated for 1 d at room temperature, and then destained in chloral hydrate solution (250 g of chloral hydrate per 1 ml of water) for 1 d followed by 95% ethyl alcohol for 2 d. Samples were then maintained in 75% ethyl alcohol prior to being photographed. To detect the development of *P. viticola* infection in grape leaves, the leaves were decolorized using 95% alcohol in boiled water for 5 min, and alcohol was removed and incubated in 0.05% aniline blue dissolved in 0.067 M K_2_HPO_4_ (pH = 9–10) for one night. The photographs were obtained by a fluorescence microscope (Olympus bx-51). The wavelengths used for excitation were 400–440 nm (blue/purple)^56,57^.

### Measurement of ion leakage

Following infiltration, leaf discs (1 cm diameter) were immersed in 5 ml of distilled water in a 50-ml sterile centrifuge tube with gentle shaking at 50 rpm for 3 h at room temperature. Ion leakage was measured with a conductivity meter (DDS-307, LeiCi, Shanghai, China). Total ion leakage was measured after incubation in a boiling water bath for 25 min. The results are expressed as a percentage of total ion leakage.

### Yeast two-hybrid screening and assays

To screen for effector-interacting proteins, a cDNA library was prepared from the susceptible *V. vinifera* cultivar ‘Pinot Noir’ inoculated with *P. viticola*. Screening was carried out using the Yeastmaker^TM^ Yeast Transformation System 2 (Clontech) according to the manufacturer’s protocol. For analyses of effector-importin-α interactions, the *P. viticola* effector sequence was inserted into the plasmid pGBK-T7 (BD), and the importin-α sequence was ligated into the plasmid pGAD-T7 (AD).

## Supplementary information

Importin-ás are required for nuclear localization and function of the Plasmopara viticola effector PvAVH53
