# Supplementary material for: Importin-αs are required for the nuclear localization and function of the Plasmopara viticola effector PvAVH53
Source: Hortic Res. 2021 Mar 1;8:46. doi: 10.1038/s41438-021-00482-6 (PMC7917100; doi:10.1038/s41438-021-00482-6)
Supplement: Supplementary file 1 — Importin-ás are required for nuclear localization and function of the Plasmopara viticola effector PvAVH53 [file 41438_2021_482_MOESM1_ESM.doc]

**Importin-αs are required for nuclear localization and function of the *Plasmopara viticola* effector PvAVH53**

**Tingting Chen^1,2,3^, Jing Peng ^1,2,3^, Xiao Yin^1,2,3^, Meijie Li^1,2,3^ , Gaoqing Xiang1,2,3 ,Yuejin Wang^1,2,3^, Yan Lei^4^, Yan Xu^1,2,3^**^*^

**Supplementary Date**


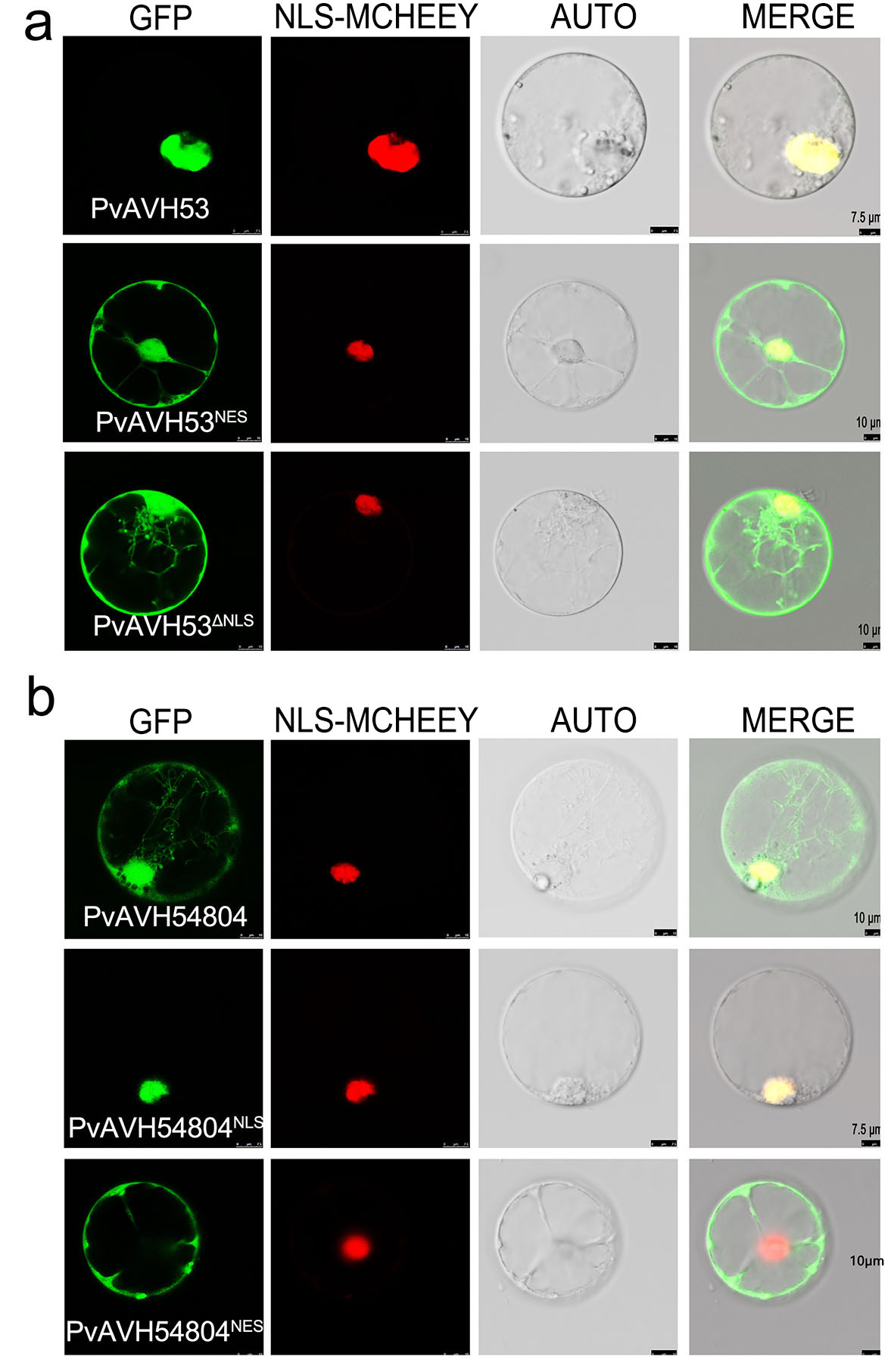


**Fig. S1 Subcellular localization of modified PvAVH53 and PvAVH54804 in *Vitis vinifera*.**

(a) Modified PvAVH53 with nuclear export signal(NES) or nuclear localization signal (NLS)deletion were co-transformed with NLS-mCheery maker in *Vitis vinifera* protoplasts showed that both the mutants of PvAVH53 failed to stabilize the nuclear localization. (b) Modified PvAVH54804 (added nuclear export signal (NES) or nuclear localization signal (NLS)) were co-transformed with NLS-mCheery maker in *V. vinifera* protoplasts. The photos were taken after protoplasts were incubated for 20-24 h in a weak light at 25℃. Scar bar=7.5-10 μm.


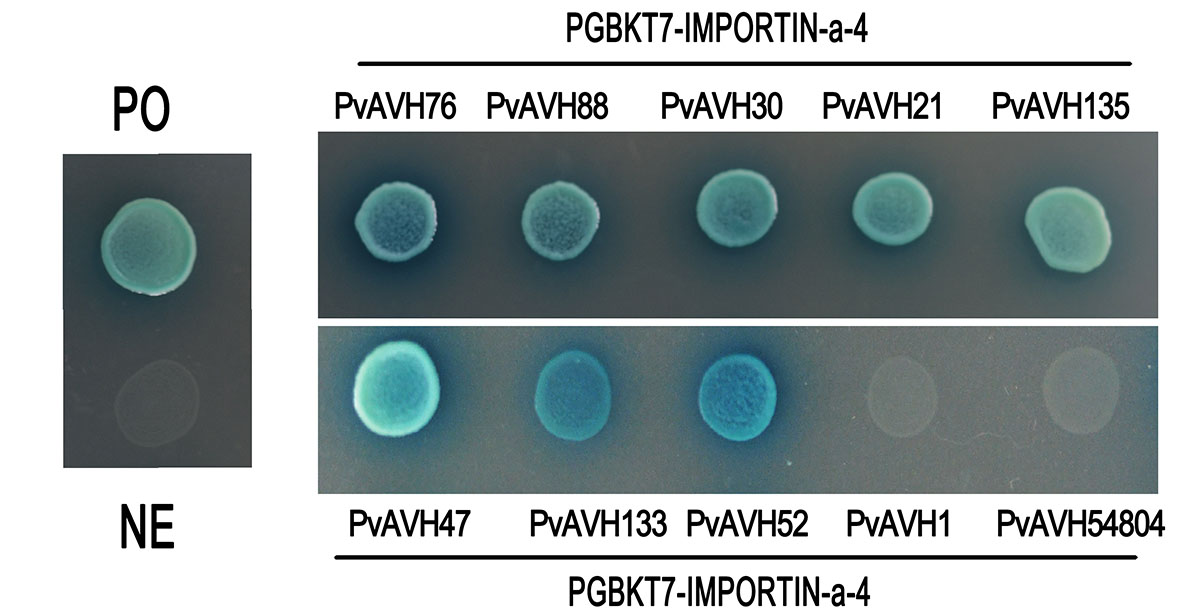


**Fig. S2 Interaction between 10 effectors and VvImpα4 were verified by Y2H assay.**

Y2H analysis showed that *Plasmopara viticola* RxLR-type effectors (only the nucleus localization and containing NLS) interact with VvImpα4. The positive colonies grew on QDO/A/X (Minimal Media Quadruple Dropouts: SD-Ade/ -His/ -Leu/ -Trp) in the presences of Aba and turn blue in the presences of X-α-Gal.


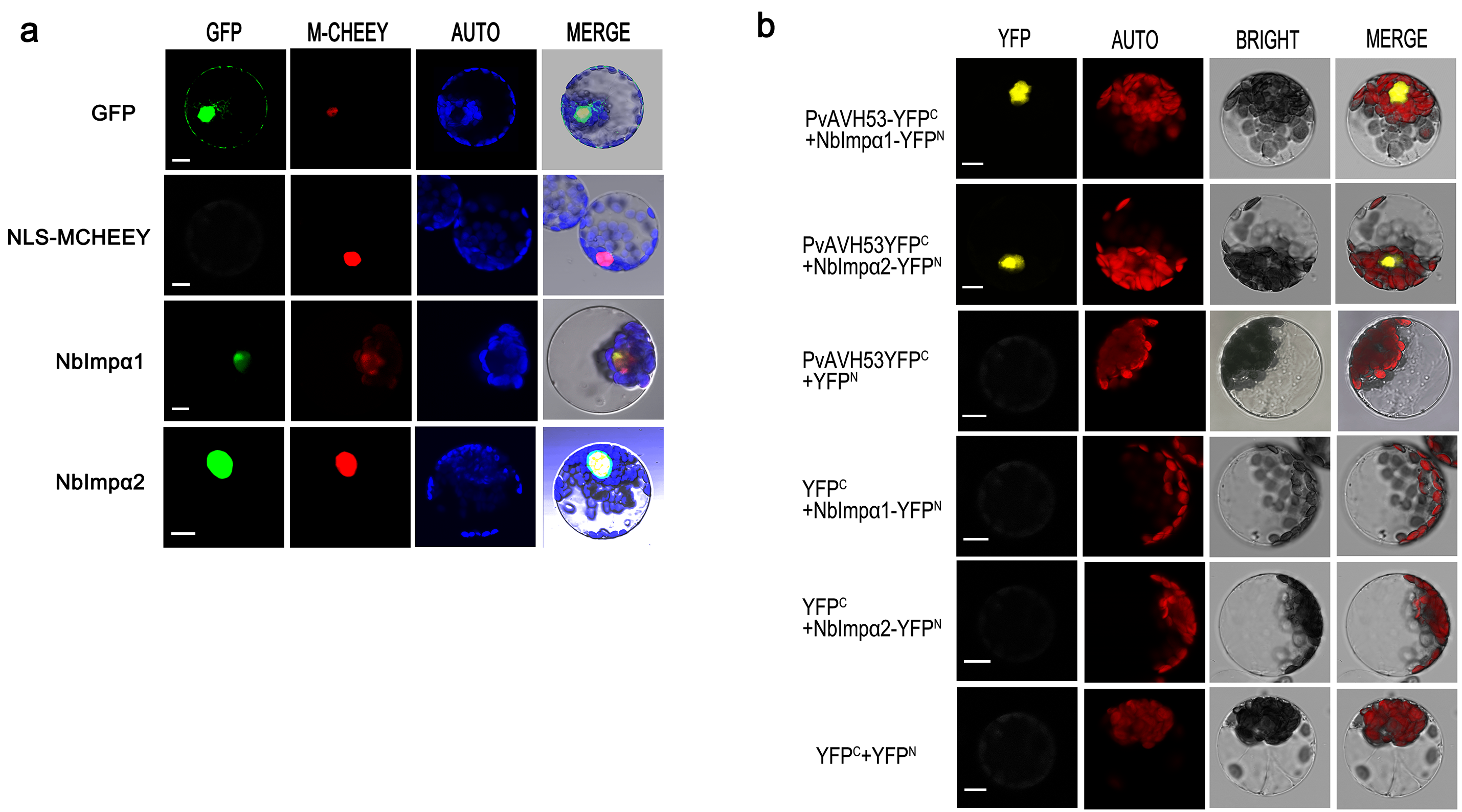


**Fig. S3 Subcellular localization of NbImpα1/2 and interaction with PvAVH53.**

(a) subcellular localization of *Nicotiana benthamiana* nuclear import factor NbImpα1/NbImpα2 and GFP as control co-transformed with NLS-mCherry in *N. benthamiana* protoplasts. (b) A BiFC assay confirmed that NbImpα1/NbImpα2 interacted with PvAVH53. The photos were taken after protoplasts were incubated for 20-24 h in a weak light at 25℃. Scar bar=10 μm.


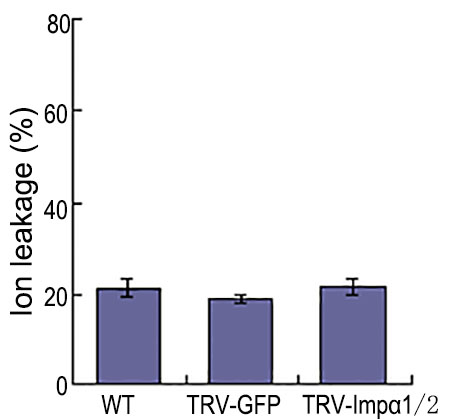


**Fig. S4 Viral-induced gene silencing (VIGS) in *Nicotiana benthamiana* has no significant difference in ion leakage.**

Ion leakage (%) measurement at tobaccos leaves (TRV-GFP, TRV-Impα1/2 silenced tobaccos and wild type tobaccos). Data are means ± SE based on three independent replicates (Student’s t test: **P < 0.01).

**
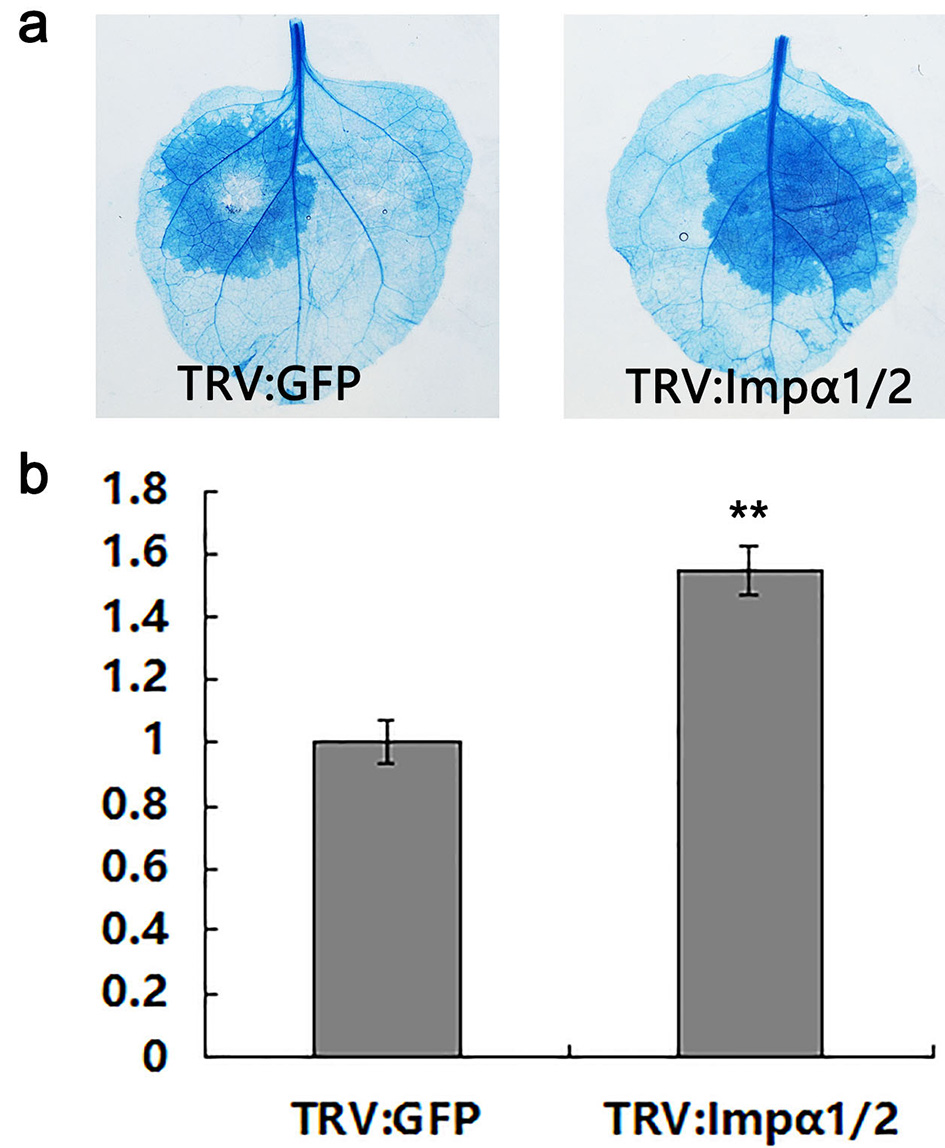
**

**Fig. S5 NbImpα1/2 promotes *Nicotiana benthamiana* susceptibility to *Phytophthora capsici*.**

(a)Lesion of control (TRV: GFP) and Impα1/2 silenced plants were inoculated with *P.capsici*. The inoculated site were staining with trypan blue. (b) Lesion diameter on *N. benthamiana* leaves measured 36 hours post inoculation. Data are means ± SE based on three independent replicates, each including 5 individual leaves (Student’ s t test: **P < 0.01).

**Table S1. Table of Sequence and accession numbers for effector genes/proteins.**

| **Effector name** | **Genbank accession number** | **Amino acid sequence（red color means NLS sequences）** |
| --- | --- | --- |
| PvAVH1 | MN328406 | MHSFKLLLALIVAICTSCDAVPRGSLSDESNFKSYPVAQYEAANHRLLRAKDGKVRADEERLSSNPDSMLTRIKSFVNPGPFHELVRTATAIAERLKETVHSTLDHWLTIQRFNHLLGHCDHGSMDSAIVRGFHPSEFRVWLDLKSPLATEVVDSLDEWPKSTQLQSLLKFIKHYHSLLLPPPNHWAKVRASINPSHASSKPLFHDVYGIKEALEEMDHIIDQDLSVATMLEQKVSPLLYKVALEARELKTGKRIDRSKLNRFIKGYMAQYPSLDKFESMVNGYAPPGKFRKIPTFKGLDDDVINPPKYLNP |
| PvAVH30 | MN328409 | MRGTLATALLLITSSRTAAESYQIDPRQLSPHVVETADKMHTTSSPRKLLRDNATNESRTIEKVITSAMHGITDPSGASTATRDTVREILDLNAFPKEAVRPRFDLNVPPSETVALLAETSEIPQQTHASTSTASDIAVSSGRTTNQRTAKPQARLGMGRNAPTRDSLVKKPSKKLAALKVTKSKNKARKYLHTSVVDAVYTMFVKHLETNSVEFDPTKEETKAILYLILEKSATPPTVSALHSNYFKQFENEDLEGLKEKLGINLMSTLSLLASLKLSSEVLNEIQRLFVWYLSLERLRAMYCFFFEFYEVDLNEIKKLLPDFRGWKVDNAARDRLFAELRKDLKHTTKLKRSLDLLRNADDVMAKHDVEEETAAAVRKAGQHFVENEHQFPSIPAPPAALHS**RPGKQHTDLSPYDLQTPVPEKNYFQHIMSND**QPRGKQPRRF |
| PvAVH76 | MN328413 | MRGAFYVTTALLLTHSVRSAAEEQQQTSHHDGAVMKSIDTESSPGKYLRDSREHHDDLGPSAVDEERTPEASTRIIENSLTLLREEALDPIRKAAYLSLDLNALPEENLTGALEAYNIVRNKDPRTSTASNPEVSSSHLSKKRSLDKASGSGLPKQV**IVHRPKKMRLSV**PLRTTDVRRSISHGKMTPMPSTVSIKDVHKLYLNHLTRTSTRYIPTRDETKKLLRLVNRIKVTRPSSNIYKPDLKNLMKMELEDLEKLFGIPKQNLGSSSEALLKLPTEDQREIKRCLYQLQGLKRWRLMYRDFFNFCLSNAAVCSNFHPQSALQVELKMKKLAESGASVANYAKVETRTTALTEAERDVLKDLKAAVERANLSETDKKAMYKETVEEVYRDDDQLTSSDLSALIADASFKQPRKRHSLVKYDALKTPLSFGTDEEALLKAQGGLLRDRKKVYELVKVVTDRTKKTNKRYDKAIYEDEFDTITDIHKKFIQDLKLRYIPTLVVTKKMWRMFNWAERPDPSKSTKVGIYLLDDVCLMDANEVAVGLGLNRAELEAALKELGADEEEALLNLKTDYLLWLLETYDKFFFFCEKNNKLCKMPSELWLTAKRATVSRTQLIKRRPLRGNHHAETS |
| PvAVH88 | MN328414 | MFLKSGFKRVACGGILLSCAFLSHGSGVAAEANATRALSARQRNLAFNQSQSDENATVAPRQVDDGDDDDDERALALFESVSKASTNAMKGIKSNAMVQKVFSKDPKLARTKSSFDEYKLQLSDPDNFFTQANFQPWFEKTIQRYKSDETKAYAAMYSTVLSRSKGDEEAVARLISSRSNLHAFHWFDAQINSWKARNLDTEEVFDLLKLSNEGPDLLNSPKLSSWVRFAGTKNEDPAERLWKYLTTRGVLKGKSDDKTPREAYSTAELGRLLQTPRETLSAVRLGPSLEKMLLVRWKQQKYPIGRALQDVGIRPPNLEVWLAYVRTLDYDNSYAIFATAGKFEVIAIAGAIGLALRSKNNLMAEAGKNMQIAQNKEWLNIDGKGLFKTYDQLFQDLKLNDAGKNLFASPLWTVWINYLKFADKASAKNTMMTILKAHFNEEELLGIAEEAEKVDSTKSIASFVKKHYFGEYRYPVSAQNVPKSSA |
| PvAVH21 | MN328417 | MRGAFYVTTALLITNSIRTAAEADRSEYRQRSYHVGVAPGSMDTKSSPTRFLRGSHEQHDDFIVSAADEERMPEASARIIGNSLTSVSEEVTGTMRKAAHLDFDLNAPPKETVTGALEAYNILRNKEPRSYTAVNAEVSNSHLSNKRTAEALTSLDKASGFAISKPLSGGQP**KKKLRTKA**PVRGTKVQSGIGHDKMTLVPRPLSEDDVQNVHKLYLKHLSRSSAKFEPTEGETRKMLGLINDTGNARPSLAKTYAPYVEELMKMNLEKMKELGLDPKTVLKPSLMELLERPSEDLREIQLCLLRLLRLKRWMRMYRNFFDFCLSRPEVCSNLSAASTLQIELKLMAEYKPSFEVASHIAVLEPEEIESTKAEREMLKRVARAIKKSNLKETAKATMYKIVGEIYRDDVHLTSLDLSAFSAGTLNIKTGKRRTYLSSDVLKTPSRFGRNEESLSGERSRDRKAITKLIEKAVNRMETTKRYDQSVKGEDEVAKLTEIRTAFIKDSHLGNELTLEMIRKMVRFSNWVEGSSDPYIVSRPGQMLKDYVAGMKIKDLETELGSNWEEFKKKTLEHLSAVQKEPKLSLLELQTDYLLMLLEAYDKFYPFCVRNPFLCKESITQRL |
| PvAVH133 | MN328422 | MRGAHYVAFALLVAASTRTAAEPDQAEPHIAPDNDYMTSGGAFNKMLPRRILRESPDPKDRLPVYASDEERMVNRLSNGNSIAKGLERTIMKAANVLRTNGEDVIANAAKPIKNYNRLRPKLEI**KKSKRQRIE**PTLSKSSEHKLHTTSNSKKSLVSSASANGQGRDEPRATVNTAKKMQHNHRSAPSRSSPTSADVSDGRLEKQLNAQKAINLDKNKRPDEAKIRNKKQHVIDPTPKNENGQALRAPPTPESLGLVANNALFASVERNEPKVTVDTATKPVKKTQHDYRSAPSSSSMTSAAVPDGQLNKQLIARKGDKVDENKHPDKVEINIKKRQRIDPKPHNVNGHDLHATSNPRKNAVLVAEDAPEVLAKKLKPNEPTFIINNAVTFLKRHDFHSARSDSFPTSAAAFDGRFNKQLIAQKPFGIDLNKYPDEVEIVSSQRQSIDRAPNNVIGQALHAPPSFEKALLSIANNAPFTSAKRLGTGELTVTVNNAVRPITEHGERSAPSASFPTSAATFDGRLNKELITEKAFKFDLNKHPDEVEFHSVEHPLHLRKGKEKAAHPATVNGGSVPINWKKFAKGPLVRKSDKLTEKVKMVHKAFLQAFSLPFHQYPTETIVMLRLVEWKTSLSPNSIHVIKSLRDLAKAQKLQRLKTLLKSDLKKLLGEEMAARENDEKVLKDAYIVKLVIMYELFYDFCHGNRDLVGNLPKKSIRRDWVLKLST |
| PvAVH135 | MN328423 | MRGAYYVAIALLVAVSCQTAAESDQAEPHQALTNHFMTSGGTGNKMLPRRILRDSHDLKDKLTVYASDEERMLNRLSSGASIAKGAEQMIKETATVLRADGENVIANAAKPIKNFNRMKPMVETQSTKRQRIIPTLNKVGGDEHHTSPKSGFPLPSFASANRRGKDELRVNVISAGSSVKGKQHEYRYTPSGSSPTSAAAADGRPNKQLKAIDLDKNKRLDEGKI**KNKKRQRIDS**MPDDEGGEALHASPNLEKSLLASASAKRLKKDEPRVNVNTVVGSVKTSQHDSRSAPSGSTPTSTAAADGRLNKQLIG**RKAKNNDKNKRSDEVKPGNKKRQRIE**PMPDDVGPHELHTTPKSKKYMVSVTKNAPVVLAKRLEPNEPTFIINNAVGYLRKHFLSAPSDSFPTSAAAPDGRLSEQLIARKHFEINLDKLPDEVEVRNSQRQSVDSMPYNLVGQALHAPPNFEKDAVSVANNAPFASANRLRIDEPTVSSNKAVRPTTHGDRPAPPSSSSTNAAAPNGRLDKQLPSQKAFKFDLNKHPDEMEFDSVERPLHLREENEKFALPAVKSQSIPINWRSEFKSGPYVRKKDRLTLKARKVHEAFIQAYNLPFHQYPAETAKMLRLVKWIKHHASPNNHHIFESLRDLASSQKLKRLKTLLRSDLENLLGNKVTELENDEKILKDAYTVKLVIMYELFYDFCHGNRNLIDNLPKESTRRDSILKVST |
| PvAVH53 | MN328426 | MRGAYYVAIALLVAASSQATAEFDQAEPQQAPNNDGVTSGGTVDKLMPRRVIRESPNAKDKLMVDAGNGERMMGHFSNEIGFSKELEQNVIPSTNGIRTGADNVIAEAAEAIERFKQLKPVLEA**LNNKRKRIDS**TPNNVGGQALHTRSIETPLVQVANIASFASAKSLKINKPNVDMQKTARIITQHDYRSAPHGYFTFRADVPDSRPNKQLIAQKALEFDLNKYPDEVEIRNSEHQRIDSMRNSVGGQAPHALPNPEKSLVSVAINPTKSLMPLTNNAETVLTKRLGGFTSKVILKNAIKSVAEHNLQHKPLVFSATIAADPVGQLNKPLIVQKALEVGNIKYPDKVNSHSANHPLHLLEGNEKSAHPATADGQSVPIDWEAEIAKGPDVGPKVVVDDKVKEIHEAFLKAFSLPFHQYPQETAKMLRPLRWKNSSPNNRVLVQSLSILATSQNQDVLMRVLGPDMTKLLGTGESALQATKVNLDAAYTAKLAIMYELFYEFYHGGGIVIDKLLSKIKSHQSIFEQ |
| PvAVH54804 | MN328431 | MYLPLYVRVLTVVALIASVDASSVNRTELIVKKSNAALGLAPTPVTRDEGFKRPLGDEQAVNDDTNGEDRFIGNIFRKNPGKVIDKPLGPVKPHEVALEAQINHWLGANLPLDEVFNRLAYGKTRQEEFLAKTVLPFYIAYSQATALKRGSQDIYGVEVLLKKFWRDDLVRLLNLGLNNRDEIGRDTARKISIGLVDWYYRKNYEAEYVANMLQGDQPILHRVHAEVIESRYNPKKAAGTTRASDVNVN |
| PvAVH47 | MN328429 | MRGTIYVAIAILVAASSRASAESDQVEPQQAPNDDFVTSDDTIYEVLPTRILRESRGSNDKLAVGAGDEERMMYNLPKGNSLSERLEQTTIKLTTDDVIAKAEEAIENFKQLEPVL**DMIRRKRQRID**PTPSNLGRQALHAPPNPDKSLVSVTENAPNVIANRLEKSGPTVIMKNAVRFIAQHDYRPAPSGSSTTSAAATDIRLHEQPIARKGSELFKNIYPNKMVSNSVEHPLHMLEGNENSAHTVTVNGITYLMAQGPALGRKDTVNEEAKKIHEAFLKAFSLPFHQYPEETTHMLRLLRWSFNSSPNNVNTATSLKDLANSQDPDVIMNLLDMDLKKLLGDGRSAVKATEKNLKEAYSVKLLIMYELFYDFCHGNKKLVGNLPSKSDRIHSILKVTT |
| PvAVH52 | MN328425 | MRGAHYVAIALLVVACGQATAGFDQAEPQQAPHNDGVTSGGNVNELLPRHVLRESPNVKDTLAVDAGSEERMVGHFSNEIGISKELDQNVISSASKIRTGADDVIAEATEAIEQYKQLKPVSKA**LNSKRKRIDP**TPNNVGGQALHTPNIETPLVPVANIASFASAKNLKKIKPNVDMQKTARIMAQHDYRPAPPGSFTFGAAVPDSRPNKQLRAQKALEFDLNKYPDEVEIGNSEHQRIDSMRNSVGGQASHALPNPEKSLVSVAINPTKSLMPLTNNARTVLAIRLGRYTSKVIVENAIKSVAVHNLQHKRLVSSATIAADPGGQLNKQLIAQKTVEFGNINYPDKVDTHLVNHPFYLLEKNEQSAHPATTMGQSVPIDWKAEIAEGPVVDFEVVVDDKVKEIHEAFLKAFSLPFHQYPQETAKMLRLLRWQNSSPKNARLLRTLKFLATSQTQDVAQRVLGPDMTKLLGTGESALQATKVNLDAAYTAKLAIMYELFYEFCHSGGVVIDELPSKVNSHQSIFEH |

**Table S2.** **Table of all primers used in this study.**

| **Vectors** | **Name** | **Primers** |
| --- | --- | --- |
| pGBK-T7 | BD-AVH53-f-Bamh1 | CCG AAT TCC CGG GGA TCC GT-ATG GAA TTT GAC CAG GCC GAG CC |
|  | BD-AVH53-r-Pst1 | TAGTTATGCGGCCGCTGCAG-TTA TTG CTC AAA AAT CGA TTG ATG GGA TTT GAT TTT C |
| pGAD-T7 | AD-AVH53-f-Bamh1 | GG CAT CGA TAC GGG ATC CAT-ATG GAA TTT GAC CAG GCC GAG CC |
|  | AD-AVH53-r-Pst1 | TAT CTA CGA TTC ATC TGC AG-TTA TTG CTC AAA AAT CGA TTG ATG GGA TTT GAT TTT C |
|  | AD-importin-a-4-f-Ecor1 | GCC ATG GAG GCC AGT GAA TTC ATG TCG CTG CGG CCC GG |
|  | AD-importin-a-4-r-Bamh1 | C AGC TCG AGC TCG ATG GAT CC TCA ACC AAA TTT AAA GCC ACC TGG AG |
| pGBK-T7 | BD-Importin-a-4-f-Ecor1 | ATG GCC ATG GAG GCC GAA TTC ATG TCG CTG CGG CCC GG |
|  | BD-Importin-a-4-r-Bamh1 | C CGC TGC AGG TCG ACG GAT CC TCA ACC AAA TTT AAA GCC ACC TGG AG |
| pGAD-T7 | AD-importin-a-f-Ecor1 | ATG GCC ATG GAG GCC GAA TTC-ATG TCT CTG AGG CCT AGC GCC |
|  | AD-importin-a-r-Bamh1 | C CGC TGC AGG TCG ACG GAT CC -TCA ACC AAA GTT GAA TCC ACC AGG AG |
| pUC-SPYNE | NeF-Import-a-4 | AGG CCT ACT AGT GGA TCC ATG TCG CTG CGG CCC GG |
|  | NeR-Import-a-4 | GGG AGC GGT ACC CTC GAG TCA ACC AAA TTT AAA GCC ACC TGG AG |
|  | NeF-Import-a-F-Xho1 | AGG CCT ACT AGT GGA TCC ATG TCG CTG CGG CCC GG |
|  | NeR-Import-a-R-Kpn1 | GGG AGC GGT ACC CTC GAG GAG ACC AAA TTT AAA GCC ACC TGG AGG AA |
| pUC-SPYCE | CeF-RXLR53 | CGC GCC ACT AGT GGA TCC ATG GAA TTT GAC CAG GCC GAG C |
|  | CeR-RXLR53 | GGG AGC GGT ACC CTC GAG TTG CTC AAA AAT CGA TTG ATG GGA TTT GA |
|  | NeF-NbImpa1-f | AGG CCT ACT AGT GGA TCC ATG TCT CTG AGA CCA AGT GCT AGG |
|  | NeF-NbImpa1-r | GGG AGC GGT ACC CTC GAG TCA ACC AAA CTT GAA TCC ACC AGA G |
|  | NeF-NbImpa2-f | AGG CCT ACT AGT GGA TCC ATG TCG CTG AGG CCG AAT TCG |
|  | NeF-NbImpa2-r | GGG AGC GGT ACC CTC GAG TCA TGA ACT GAA GTT GAA TCC TCC TGA |
| pGAD-T7 | AD-AVH21-f-Ecor1 | GCC ATG GAG GCC AGT GAA TTC-ATG GAT CGA TCG GAA TAC CGA CAA AG |
|  | AD-AVH21-r-Bamh1 | C AGC TCG AGC TCG ATG GAT CC-TTA GAG CCG CTG AGT AAT TGA CTC C |
|  | AD-AVH133-f-Ecor1 | GCC ATG GAG GCC AGT GAA TTC-ATG GAG CCT GAC CAG GCC G |
|  | AD-AVH133-r-Bamh1 | C AGC TCG AGC TCG ATG GAT CC-TCA AGT TGA AAG CTT GAG GAC CCA ATC |
|  | AD-AVH135-f-Ecor1 | GCC ATG GAG GCC AGT GAA TTC-ATG GAC CAG GCC GAG CCT C |
|  | AD-AVH135-r-Bamh1 | C AGC TCG AGC TCG ATG GAT CC-TTA AGT CGA TAC CTT CAG GAT CGA ATC |
|  | AD-AVH76-f-Ecor1 | GCC ATG GAG GCC AGT GAA TTC-ATG GCC GCC GAG GAA CAA CAA C |
|  | AD-AVH76-r-Bamh1 | C AGC TCG AGC TCG ATG GAT CC-TCA AGA AGT TTC TGC GTG ATG GTT TCC |
|  | AD-AVH88-f-Ecor1 | GCC ATG GAG GCC AGT GAA TTC-ATG GAG GCC AAT GCG ACG CG |
|  | AD-AVH88-r-Bamh1 | C AGC TCG AGC TCG ATG GAT CC-TCA AGC TGA CGA TTT AGG CAC ATT CTG |
|  | AD-AVH47-f-Ecor1 | GCC ATG GAG GCC AGT GAA TTC-ATG GAC AAG CCA CGC CCT GAA |
|  | AD-AVH47-r-Bamh1 | C AGC TCG AGC TCG ATG GAT CC-TCA ATT GGT GGA GAA GGT GGT ACA G |
|  | AD-AVH30-f-Ecor1 | GCC ATG GAG GCC AGT GAA TTC-ATG GAG TCT TAC CAA ATT GAT CCT CG |
|  | AD-AVH30-r-Bamh1 | C AGC TCG AGC TCG ATG GAT CC-TTA AAA GCG TCG AGG TTG CTT TCC TC |
|  | AD-AVH52-f-Ecor1 | GCC ATG GAG GCC AGT GAA TTC-ATG ACT GCA GGC TTT GAC CAG GCC |
|  | AD-AVH52-r-Bamh1 | C AGC TCG AGC TCG ATG GAT CC-ATG CTC AAA AAT CGA TTG ATG GGA GTT GAC |
|  | AD-AVH1-f-Ecor1 | GCC ATG GAG GCC AGT GAA TTC-ATG GTC CCA CGT GGC TCT CTG |
|  | AD-AVH1-r-Bamh1 | C AGC TCG AGC TCG ATG GAT CC-TCA CGG ATT CAG GTA TTT CGG AGG |
|  | AD-54804-f-Ecor1 | GCC ATG GAG GCC AGT GAA TTC-ATG TCT TCC GTG AAT AGA ACA GAG TTG ATT GT |
|  | AD-54804-r-Bamh1 | C AGC TCG AGC TCG ATG GAT CC-TTA ATT TAC ATT GAC ATC GCT AGC TCG TGT |
| Pcambia2300 | GFP-PvAVH53-Sma1-f | gagctc ATG GAA TTT GAC CAG GCC GAG CCT C |
|  | GFP-PvAVH53-Not1-r | TTGCGGCCGCAA TTG CTC AAA AAT CGA TTG ATG GGA TTT GAT TTT C |
|  | GFP-PvAVH54804-Sma1-f | GAGCTC-ATG TCT TCC GTG AAT AGA ACA GAG TTG ATT |
|  | GFP-PvAVH54804-Not1-r | TTGCGGCCGCAA-ATT TAC ATT GAC ATC GCT AGC TCG T |
|  | 54804GFP-NES-bamh1-r | TGGTGTCGACTCTAGAGGATCCcttgttaatatcaagaccagcgagctttaatgcaagctcgttATTTACATTGACATCGCTAGCTCGT |
|  | 54804GFP-NlS-bamh1-r | TGGTGTCGACTCTAGAGGATCCAACCTTACGCTTCTTTTTAGGATTTACATTGACATCGCTAGCTCGT |
|  | RxLR53GFP-NES-bamh1-r | TGGTGTCGACTCTAGAGGATCCcttgttaatatcaagaccagcgagctttaatgcaagctcgttTTGCTCAAAAATCGATTGATGGGATTTG |
|  | EF1a qPCR F | CTGGATTCGAGGGAGACAACA |
|  | EF1a qPCR R | GCACCGTTCCAATACCACCAA |
|  | nt-TRV-Impa1-f-RQ | ATGTCCGTGAGCAGGCTGTG |
|  | nt-TRV-Impa1-R-RQ | GGAGGTTGTGGCTTGCCTCT |
|  | nt-TRV-Impa2-f-RQ | CCAAGCAGCTTCAAACTGGA |
|  | nt-TRV-Impa2-R-RQ | TGCAGCTGGAGGCCACCACA |
|  | Vvimport-a-4-rq-f | CCCGGCAACAGTACTCAACG |
|  | Vvimport-a-4-rq-r | CCCGTCTTGTGCAGTTCCTTG |
|  | Vvimport-a-rq-f | CAGCCGCAGGTACTCAAACG |
|  | Vvimport-a-rq-r | ACCCTGCCCTATCTCCTCCA |
| pTRV2 | TRV-NbImpa1F-Ecor1 | TGAGTAAGGTTACCGAATTCAGAGAAGAGAGTTTGCTCAAGAAGCGT |
|  | TRV-NbImpa1R-Bamh1 | GTGAGCTCGGTACGGATCCAAAGGTAAGAAAGTGCCCAGCATGCATCTGT |
|  | BD-AVH53-100aa-f-Ecor1 | ATG GCC ATG GAG GCC GAA TTC-ATG GCA GAT AAT GTG ATT GCA GAA GCT G |
|  | BD-AVH53-145aa-R | C CGC TGC AGG TCG ACG GAT CC -CTAGAGGTGTTTCTATACTTCGCGT |
|  | BD-△IBB-f-Ecor1 | ATG GCC ATG GAG GCC GAA TTC ATG TCG CTG CGG CCC GG |
|  | BD-△IBB-r-Bamh1 | C CGC TGC AGG TCG ACG GAT CC -AGT GGT TGC TTC CAG TTG TGA AGC |
|  | BD-△Arm-f-Ecor1 | ATG GCC ATG GAG GCC GAA TTC-ATG CAA TTC AGA AAG CTA CTA TCT ATT GAG CG |
|  | BD-△Arm-r-Bamh1 | C CGC TGC AGG TCG ACG GAT CC -TCA CTT CAG AAT GTT CTC AAG GCC CTC |
|  | BD-△Arm3-f-Ecor1 | ATG GCC ATG GAG GCC GAA TTC-ATG GTG GGT GAG GCT GAC AAA GAA TC |
|  | BD-△Arm3-r-Bamh1 | C CGC TGC AGG TCG ACG GAT CC -TCA ACC AAA TTT AAA GCC ACC TGG AG |
|  | pCR11-IMa-4-1-F | AAAC CTC CTG TCG TGG CAA GCC ACC AAC ACC A |
|  | pCR11-IMa-4-1-R | AAAA TGGTGTTGGTGGCTTGCCACGACAGGAG |
|  | pCR11-IMa--1-F | AAAC AAATGATGAAGAAGTCCTTACTGA |
|  | pCR11-IMa--1-R | AAAA TCA GTA AGG ACT TCT TCA TCA TTT |
